# Nanoparticle‐Mediated Immunometabolic‐Epigenetic Remodeling Enhances Schwann Cell‐Macrophage Interaction for Sciatic Nerve Regeneration

**DOI:** 10.1002/advs.202522093

**Published:** 2026-02-20

**Authors:** Wenying Xu, Rongrong Wu, Zhicheng Liu, Hongyu Yan, Huan Zhang, Yuanyi Zheng, Xiaojun Cai

**Affiliations:** ^1^ Shanghai Key Laboratory of Neuro‐Ultrasound for Diagnosis and Treatment Shanghai Sixth People's Hospital Affiliated to Shanghai Jiao Tong University School of Medicine Shanghai China; ^2^ Department of Ultrasound in Medicine Shanghai Sixth People's Hospital Affiliated to Shanghai Jiao Tong University School of Medicine Shanghai China; ^3^ Hepatic Surgery Center Tongji Hospital Tongji Medical College Huazhong University of Science and Technology Wuhan China

**Keywords:** bioactive nanomaterials, immunometabolic, nanomedicine, nerve regeneration, sciatic nerve injury

## Abstract

Peripheral nerve regeneration continues to pose a significant clinical challenge, primarily attributable to the inherently limited regenerative capacity of axons and the intricate inflammatory microenvironment that develops following injury. While immunometabolic modulation has emerged as a promising therapeutic avenue, achieving precise and sustained intervention within the injury microenvironment remains technically challenging. Here, we introduce a biomimetic Prussian White nanoparticle (PW) that facilitates long‐term local retention and drives coordinated immunometabolic‐epigenetic remodeling to promote sciatic nerve regeneration. Through integrated multi‐omics analyses, we identify a previously unrecognized S100a4^+^ macrophage substate, which is epigenetically activated via PW‐induced accumulation of α‐ketoglutarate and subsequent Kdm4a/b‐mediated demethylation of the repressive histone mark H3K9me3 at the S100a4 gene locus. Furthermore, these reprogrammed macrophages secrete itaconate, a previously unidentified neuro‐immune mediator, which effectively supports Schwann cell proliferation under inflammatory stress. This nanoparticle‐enabled metabolic‐epigenetic dialogue between macrophages and Schwann cells markedly enhances functional and structural recovery in both rodent and canine models of sciatic nerve injury. Our findings establish a paradigm of material‐mediated cell reprogramming via coordinated immunometabolic‐epigenetic remodeling, offering a versatile and translatable strategy with broad potential for treating neurodegenerative disorders.

## Graphical abstract

A biomimetic Prussian White nanoparticle (PW) is engineered to achieve long‐term local retention and orchestrate immunometabolic‐epigenetic remodeling for sciatic nerve regeneration. PW directly targets hexokinase 2 to suppress glycolysis, thereby elevating α‐ketoglutarate and driving Kdm4a/b‐mediated demethylation of H3K9me3. This epigenetic activation polarizes macrophages toward a pro‐regenerative S100a4^+^ phenotype that secretes itaconate, which in turn enhances Schwann cell proliferation under inflammatory stress, leading to robust functional recovery.

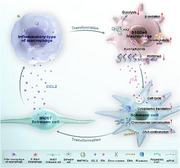

## 1 Introduction

Peripheral nerve damage can lead to severe sensory, motor, or autonomic dysfunction, and is a major challenge that clinical treatment strives to solve [1, 2]. Accelerating axonal regeneration is critical for functional recovery, yet effective therapeutic strategies remain elusive [3]. Immunometabolism, the regulation of immune cell behavior through metabolic modulation, has emerged as a promising therapeutic avenue, offering novel strategies to modulate immune responses and promote tissue repair [4, 5, 6]. Recent advancements in immunometabolic modulators, including small molecules, proteins, and antibodies, have demonstrated significant therapeutic potential [7, 8]. However, their clinical application is often hindered by rapid degradation and limited bioavailability, restricting sustained therapeutic efficacy at the target site, which underscores the need for nanotechnology‐driven solutions [9]. In this context, nanomaterials have attracted increasing attention due to their intrinsic bioactivity, tunable physicochemical properties, and potential to modulate immune responses [10, 11, 12]. Their ability to penetrate biological barriers and maintain stability within complex microenvironments makes them ideal candidates for facilitating targeted intercellular communication and sustained localized therapy [13, 14, 15]. Nevertheless, whether nanomaterials can precisely orchestrate immunometabolic crosstalk to enhance peripheral nerve regeneration, such as in sciatic nerve injury (SNI), remains an open and compelling question.

Despite the growing interest in immunometabolic interventions, the precise mechanisms by which metabolic crosstalk between immune cells and Schwann cells influence nerve repair remain insufficiently explored. The immune system plays a critical role in maintaining homeostasis and clearing cellular debris [16], with macrophages serving as key mediators of regeneration through intricate interactions with the extracellular matrix and immune components [17, 18]. Beyond classical ligand‐receptor signaling, small metabolites function as diffusible messengers within the microenvironment, shaping immune responses and cellular behavior [3, 19, 20]. Schwann cells, the main glial cells of the peripheral nervous, are essential for axonal regeneration [21], myelin clearance [22], and dedifferentiation [23]. However, inflammation‐driven recruitment of immune cells, particularly macrophages, can impede Schwann cell reprogramming, thereby compromising nerve repair [24]. Recent evidence [25, 26, 5] further suggests that metabolic reprogramming in immune cells is closely linked to epigenetic modifications, which in turn regulate cellular plasticity and functional polarization. While macrophage heterogeneity and metabolism are increasingly implicated in nerve regeneration, the specific metabolic dialogue between macrophages and Schwann cells, and its regulation through epigenetic mechanisms, remains poorly understood.

**FIGURE 1:**
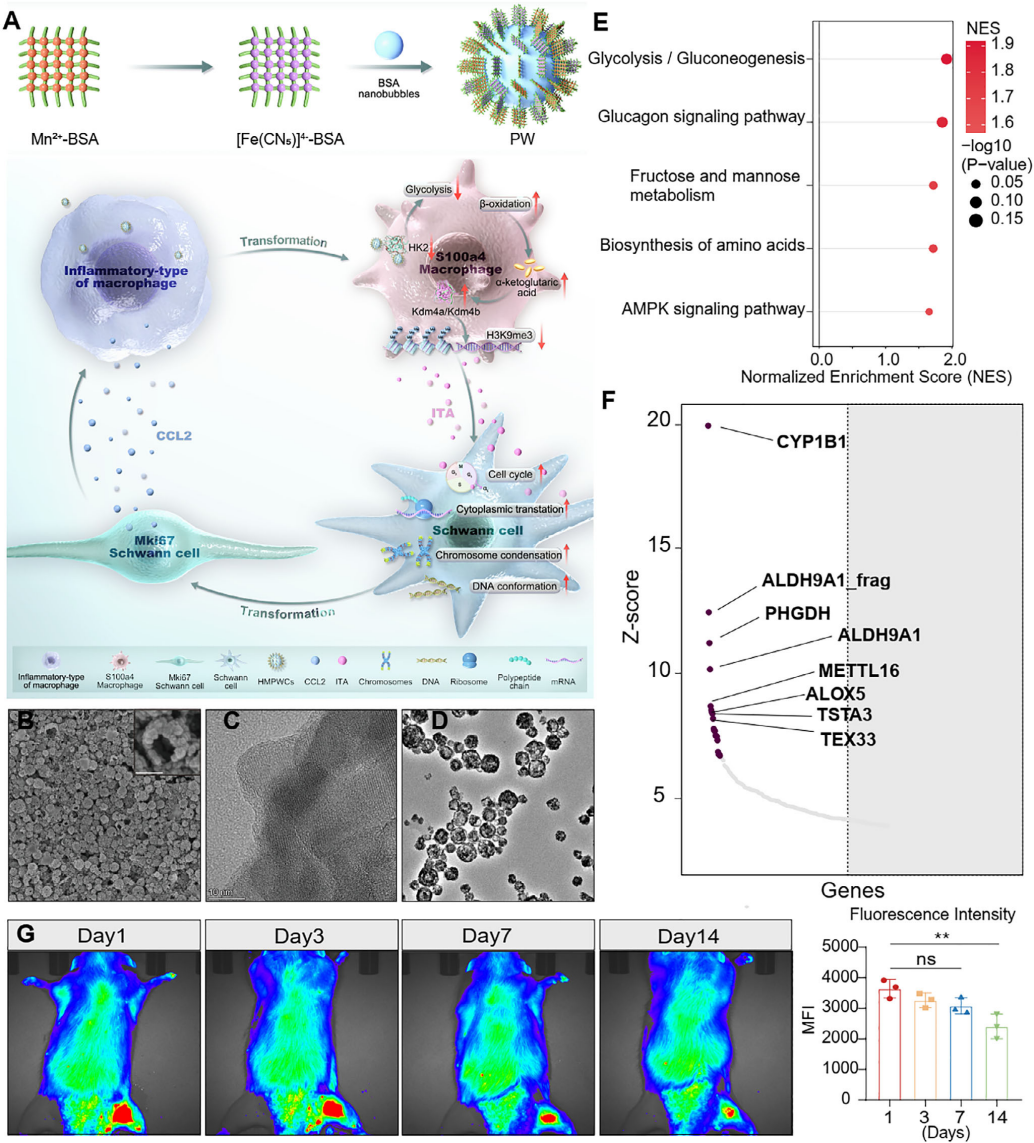
Schematic illustration and functional validation of Prussian White nanoparticle (PW)‐mediated immunometabolic‐epigenetic remodeling for sciatic nerve regeneration. (A) Schematic representation of the proposed mechanism: PW nanoparticles facilitate sustained local retention and drive coordinated immunometabolic‐epigenetic reprogramming, enhancing Schwann cell‐macrophage crosstalk to promote nerve repair. (B) Scanning electron microscopy (SEM) image of PW revealing uniform nanocapsule morphology. Inset shows a higher magnification view highlighting structural details. (C,D) Transmission electron microscopy (TEM) images displaying the internal lattice structure (C) and homogeneous dispersion (D) of PW. (E,F) Pathway enrichment analysis from human microarray proteomic profiling reveals significant inhibition of glycolysis/gluconeogenesis, AMPK signaling, and amino acid biosynthesis pathways by PW. (G) In vivo fluorescence imaging and quantification of Cy5.5‐labeled PW at the sciatic nerve injury site over 14 days (*n* = 3/group). Mean values are shown, and error bars represent ± s.d., as analyzed by one‐way ANOVA with Tukey's post hoc tests in (D). ns, not significant, ^**^ *p* <0.01.

Herein, we engineered a biomimetic Prussian White nanoparticle (PW) through a human microarray‐based proteomic screening approach to identify candidates with immunometabolic regulatory potential. Using both rodent and canine SNI models, we demonstrated that PW enables long‐term retention at the injury site and significantly enhances functional and structural recovery. Through integrated multi‐omics approaches, including single‐cell RNA sequencing, metabolomics, proteomics, and epigenomic profiling, we identified a previously unrecognized S100a4^+^ macrophage substate that plays a pivotal role in nerve repair. We further elucidated a novel mechanism wherein PW inhibits glycolysis via hexokinase 2 (HK2) targeting, promotes fatty acid β‐oxidation (FAO), and increases intracellular α‐ketoglutarate (α‐KG) levels, leading to Kdm4a/b‐mediated demethylation of H3K9me3 at the S100a4 locus. This epigenetic activation drives S100a4^+^ macrophage polarization and secretion of itaconate (ITA), a previously unidentified neuro‐immune mediator that enhances Schwann cell proliferation under inflammatory stress (Figure 1A). Our work establishes a nanomaterial‐based strategy for immunometabolic‐epigenetic remodeling that restores macrophage‐Schwann cell communication and promotes nerve regeneration. This approach not only provides insights into the metabolic and epigenetic regulation of nerve repair but also offers a versatile platform with broad implications for the treatment of neurodegenerative diseases.

## 2 Results

### 2.1 Construction and Characterization of PW With Immunometabolic Regulatory Potential and Sustained In Situ Retention

The strategic design of nanomaterials capable of modulating local immunometabolism represents a promising yet challenging frontier in regenerative medicine. To address the limitations of conventional immunomodulators, such as rapid degradation and poor bioavailability, we developed biomimetic PW via a protein‐mediated biomineralization approach (Figure 1A). This synthesis leverages bovine serum albumin (BSA) as a stabilizing and structure‐directing agent, facilitating the formation of uniform nanocapsules with controlled physicochemical properties. Comprehensive characterization confirmed the successful fabrication of PW: scanning electron microscopy (SEM) revealed a homogeneous nanocapsule morphology with an average diameter of 200 nm (Figure 1B), while high‐resolution transmission electron microscopy (TEM) displayed well‐defined lattice fringes, indicative of high crystallinity (Figure 1C,D). Elemental mapping demonstrated a uniform distribution of C, N, O, Fe, S, K, and Mn, with Fe and Mn enriched in the core region, corroborating the intended PW composition (Figure S1A,B), and X‐ray photoelectron spectroscopy (XPS) verified the coexistence of Fe^2+^/Fe^3+^ and Mn^2+^ oxidation states (Figure S1C,D). Fourier‐transform infrared (FTIR) spectroscopy identified characteristic ─C≡N─ stretches from the Prussian White framework and amide (CO‐NH) bands from BSA, confirming successful hybridization (Figure S1E). X‐ray diffraction (XRD) and Raman spectroscopy further validated the crystalline structure and cyanide vibrational modes, respectively (Figure S1F,G). Dynamic light scattering (DLS) and zeta potential measurements indicated a narrow size distribution (≈200 nm) and a slightly negative surface charge (−10 mV), supporting colloidal stability (Figure S1H,I). Critically, cytotoxicity assessments via CCK‐8 assays confirmed that PW concentrations up to 60 µg/mL were non‐toxic to key nerve injury‐associated cells, including bone marrow‐derived macrophages (BMDMs), Schwann cells, and PC12 cells, ensuring biocompatibility for subsequent biological applications (Figure S2A–C).

Beyond structural and safety validation, we sought to elucidate the intrinsic bioactivity of PW. Employing Human Microarray Proteomic Analysis, a high‐throughput platform profiling thousands of proteins, we compared PW‐biotin‐treated microarrays with biotin‐only controls to identify preferential protein associations induced by PW. Strikingly, the five most significantly inhibited pathways were all metabolism‐related, including glycolysis/gluconeogenesis and fructose/mannose metabolism (Figure 1E,F). Notably, the AMPK signaling pathway, a master regulator of cellular energy homeostasis, was also substantially suppressed, highlighting PW's inherent immunometabolic regulatory potential. This unbiased proteomic screening thus positions PW as a novel nanomodulator of cellular metabolism, independent of conventional drug loading. Given its metabolic influence, we next evaluated whether PW could achieve sustained localization at injury sites, a critical requirement for effective nerve regeneration. Using a rat SNI model, we administered Cy5.5‐labeled PW and monitored its retention via in vivo fluorescence imaging. Remarkably, robust fluorescence signals were maintained at the injury site over 7 days, with detectable signal persisting for up to 14 days (Figure 1G). This prolonged retention starkly contrasts with the short half‐lives of neurotrophic factors and small‐molecule drugs, underscoring a key advantage of nanomaterial‐based delivery systems. Collectively, PW may be served as a structurally well‐defined, biocompatible, and metabolically active nanomaterial. Its rational design enables not only long‐term in situ retention but also intrinsic modulation of key immunometabolic pathways. These properties collectively provide a solid foundation for leveraging PW in immunometabolic reprogramming within the challenging microenvironment of nerve injury, offering a robust and translatable platform for advanced regenerative therapies.

### 2.2 PW Accelerates Functional Recovery and Anatomical Reconstruction After SNI

Following the confirmation of PW's immunometabolic regulatory potential and sustained retention at the injury site, a comprehensive assessment of its therapeutic efficacy in a rat SNI model was conducted. Initial in vivo biosafety evaluations revealed no significant signs of organ toxicity or systemic adverse effects, affirming the biocompatibility of PW for further therapeutic applications (Figure S2D–G). To determine the optimal therapeutic dosage, axonal regeneration was evaluated 14 days post‐injury across varying concentrations of PW. Notably, concentrations of 2.5 µg/mL and 5 µg/mL significantly enhanced axonal regrowth beyond the injury site, whereas a higher concentration (30 µg/mL) did not yield comparable regenerative benefits (Figure S3). Consequently, a concentration of 5 µg/mL was selected for all subsequent experiments to maximize therapeutic efficacy while minimizing potential off‐target effects.

**FIGURE 2:**
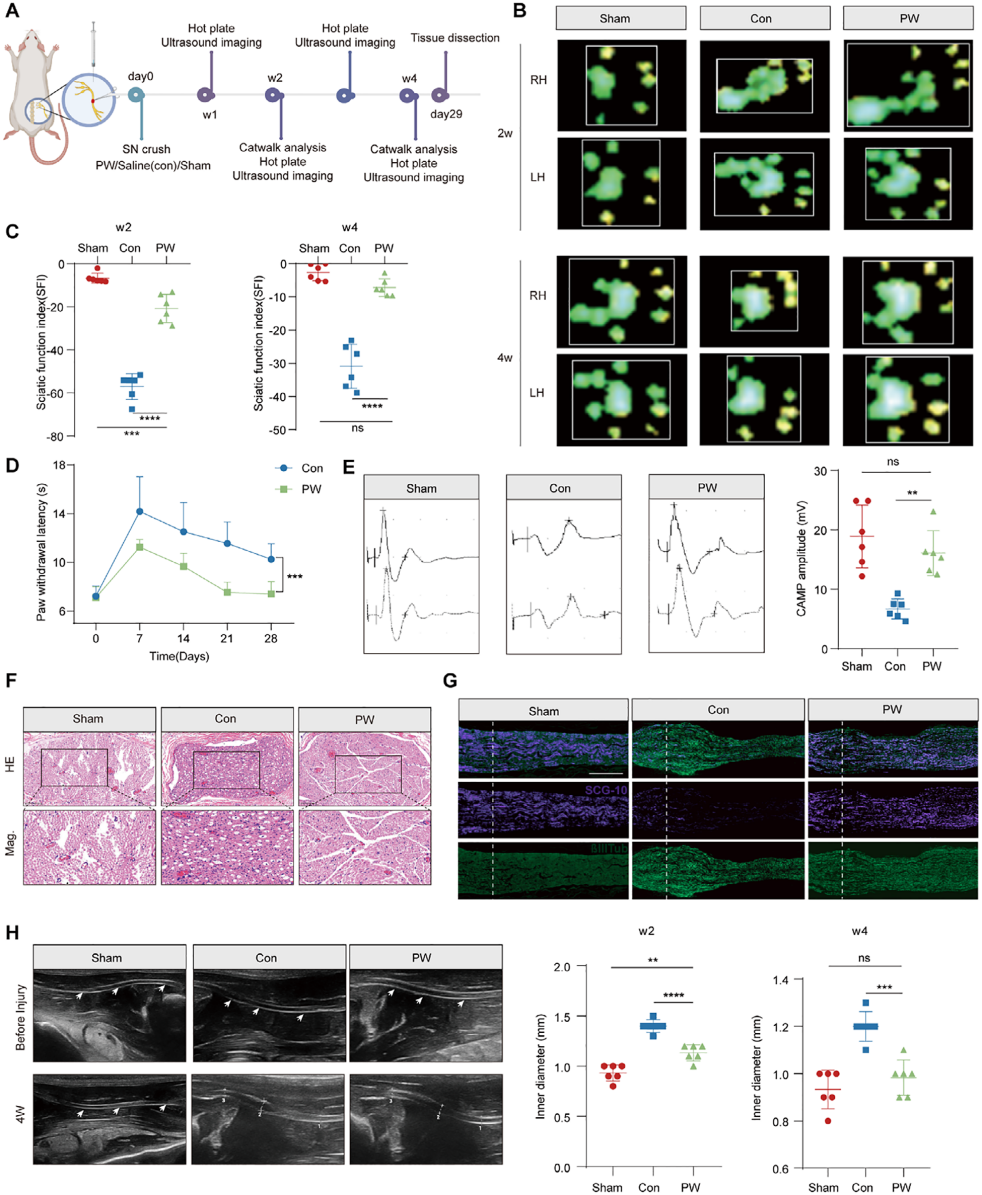
PW promotes functional and structural recovery after sciatic nerve injury (SNI). (A) Experimental timeline and schematic of the SNI model and PW administration. (B) Representative paw prints from Sham, Control (Con), and PW‐treated rats at 2 and 4 weeks post‐injury. (C) Sciatic Functional Index (SFI) scores showing improved motor function in the PW group (*n* = 6/group). (D) Paw withdrawal latency in the hot plate test, indicating accelerated sensory recovery in PW‐treated rats (*n* = 7/group). (E) Representative compound muscle action potential (CMAP) traces and quantification, reflecting enhanced neuromuscular transmission (*n* = 6/group). (F) H&E staining of sciatic nerve sections showing reduced fibrosis and improved tissue integrity in the PW group (*n* = 6/group). Scale bars: 100 µm (upper), 50 µm (lower). (G) Immunofluorescence staining for βIII‐tubulin (green) and SCG‐10 (purple), indicating axonal preservation and regeneration (*n* = 6/group). Scale bar: 500 µm. (H) Ultrasonographic images and quantitative analysis of longitudinal nerve diameter, demonstrating structural recovery (*n* = 6/group). Mean values are shown, and error bars represent ± s.d., as analyzed by one‐way ANOVA with Tukey's post hoc tests in (C), (E) and (H) or unpaired Student's *t* test in (D). ns, not significant, ^**^ *p* <0.01, ^***^ *p* <0.001, ^****^ *p* <0.0001.

Longitudinal functional recovery was monitored over a 4‐week period using a multimodal assessment approach (Figure 2A). Gait analysis, a sensitive indicator of motor functional restoration, demonstrated that PW‐treated animals exhibited significant improvements in stride length and toe extension, two parameters critically impaired following SNI. These enhancements were evident as early as 2 weeks post‐injury and became more pronounced by the 4‐week endpoint, closely mirroring the sham group's performance (Figure 2B,C; Figure S4). Quantitative evaluation using the sciatic functional index (SFI), a validated metric for motor nerve recovery, further corroborated these findings, with the PW group achieving markedly superior scores compared to the control group (−7.2 vs. −30.9; Figure 2C). Sensory functional restoration, assessed via the hot plate test, also revealed accelerated normalization of nociceptive responses in PW‐treated rats, as indicated by significantly reduced withdrawal latencies (7.4 s vs. 10.22 s; Figure 2D). These functional improvements were paralleled by robust anatomical and physiological recovery. Gross morphological and gravimetric analyses of the gastrocnemius muscle, a key target of sciatic innervation, revealed significant mitigation of denervation‐induced atrophy in the PW group, underscoring successful muscle reinnervation (Figure S5A,B). Histological examination further confirmed the preservation of muscle architecture and reduction in fibrotic degeneration in PW‐treated animals (Figure S5C,D).

Electrophysiological assessments provided additional mechanistic insights into functional recovery. Compound muscle action potential (CMAP) amplitudes, a direct measure of neuromuscular transmission integrity, were significantly higher in the PW group compared to controls, indicative of enhanced axonal conductivity and synaptic efficacy (Figure 2E). Structural evaluation of regenerated nerves via hematoxylin‐eosin (H&E) and Luxol Fast Blue (LFB) staining revealed a more organized tissue architecture, reduced vacuolation, and increased density of myelinated axons in PW‐treated specimens (Figure 2F; Figure S6A). Multiplex immunohistochemistry (mIHC) further demonstrated upregulation of regeneration‐associated markers such as SCG‐10 and NF200, both in longitudinal and cross‐sectional analyses, highlighting PW's role in promoting axonal preservation and outgrowth (Figure 2G; Figure S6B,C). Ultrastructural examination using TEM, considered the gold standard for myelin and axonal assessment, revealed significant improvements in myelin sheath thickness and g‐ratio, both of which are hallmarks of mature and functional nerve regeneration (Figure S6D–F). Complementing these histological findings, real‐time ultrasonography provided non‐invasive, dynamic evidence of nerve repair, showing enhanced epineurial continuity, normalized internal echogenicity, together with a measurable decrease in nerve size, characterized by reductions in both longitudinal inner diameter and cross‐sectional area, consistent with alleviated edema and improved structural consolidation in PW‐treated nerves. Figure 2H; Figure S7A–C). Collectively, these results demonstrate that PW not only accelerates functional recovery but also orchestrates comprehensive anatomical and ultrastructural restoration of the injured sciatic nerve. By integrating multimodal validation encompassing behavioral and electrophysiological readouts, in vivo imaging, and molecular histology, this study establishes PW as a potent nanotherapeutic agent capable of bridging the gap between immunometabolic modulation and functional nerve regeneration.

### 2.3 Single‐Cell Transcriptomics Unveils PW‐Induced S100a4^+^ Macrophage Polarization and ITA‐Mediated Schwann Cell Proliferation

**FIGURE 3:**
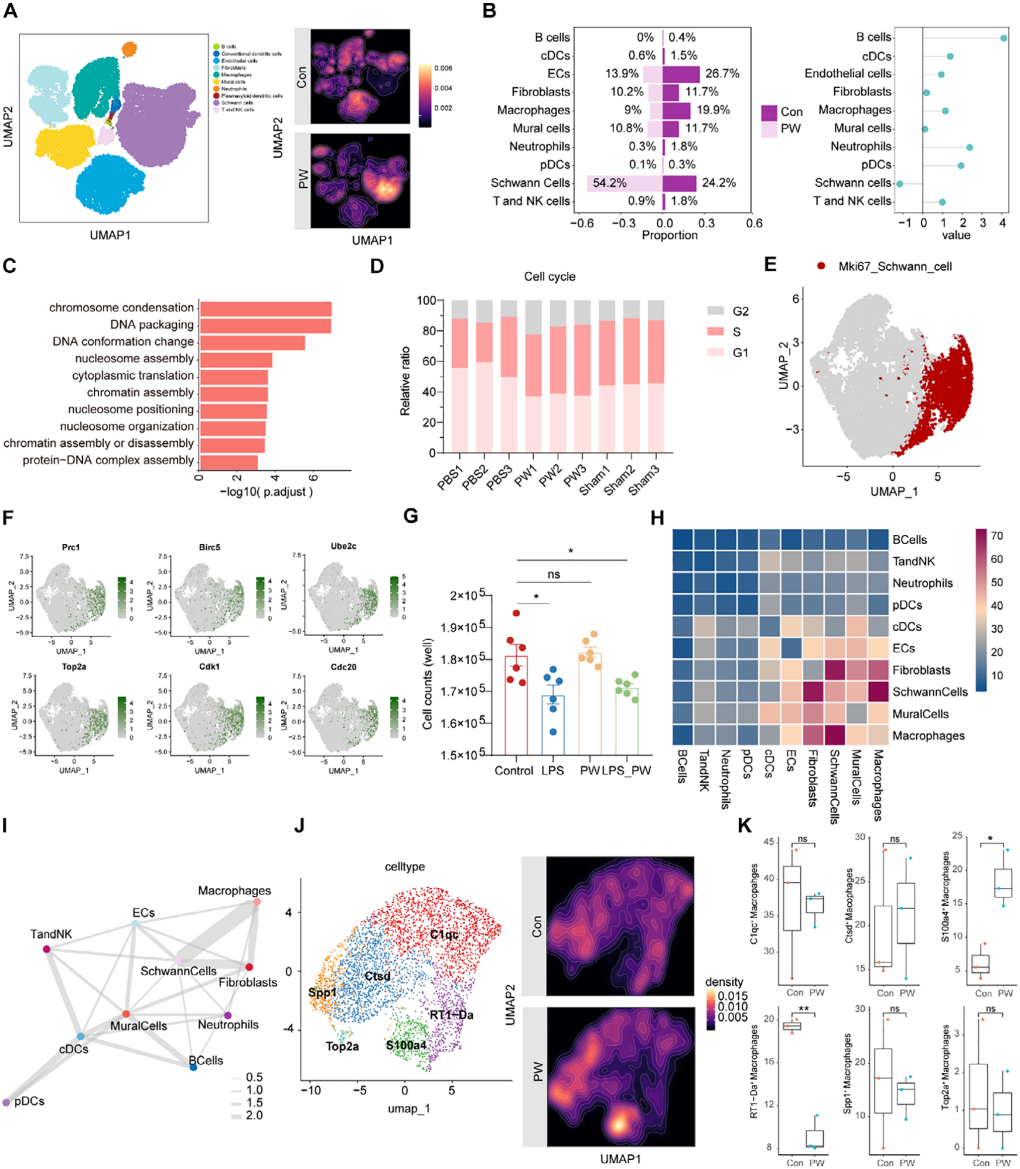
Single‐cell RNA sequencing reveals PW‐induced S100a4^+^ macrophage polarization and Schwann cell proliferation. (A) UMAP visualization of cellular composition in sciatic nerve tissues from PBS‐ and PW‐treated groups (*n* = 3/group). (B) Relative abundance of major cell types from scRNA‐seq data. (C) Gene Ontology (GO) enrichment analysis of DNA packaging and chromatin organization pathways in Schwann cells from PW‐treated nerves. (D) Flow cytometry analysis of cell cycle distribution in sorted Schwann cells. (E) UMAP plot highlighting Mki67^+^ proliferating Schwann cells. (F) Feature plots showing expression of key genes associated with Schwann cell states. (G) Quantification of total Schwann cell counts under different culture conditions (*n* = 6/group). (H) Heatmap of intercellular interaction frequencies within the sciatic nerve microenvironment. (I) Cell–cell communication network among Schwann cells, macrophages, and immune cells. (J) UMAP visualization of macrophage subpopulations in Control and PW groups. (K) Quantification of macrophage subset proportions (*n* = 3/group). Mean values are shown, and error bars represent ± s.d., as analyzed by one‐way ANOVA with Tukey's post hoc tests in (G) or unpaired Student's t test in (K). ns, not significant, ^*^ *p* < 0.05, ^**^ *p* < 0.01.

To elucidate the cellular dynamics underpinning PW‐mediated nerve repair, we performed single‐cell RNA sequencing (scRNA‐seq) on sciatic nerve tissues following injury. Unsupervised clustering identified 10 distinct cell populations, including Schwann cells, macrophages, fibroblasts, and endothelial cells (Figure 3A; Figure S8). Notably, PW treatment induced a profound shift in cellular composition: the proportion of Schwann cells increased by 2.2‐fold, while the macrophage population decreased by 55% (Figure 3B). This reconfiguration aligns with the therapeutic efficacy of PW, suggesting a pivotal role for Schwann cells in the regenerative process. Enrichment analysis further revealed that Schwann cells in the PW group were significantly associated with pathways governing cell cycle progression, cytoplasmic translation, chromosome condensation, and DNA conformational changes (Figure 3C). Subsequent cell cycle analysis of sorted Schwann cells demonstrated a marked reduction in the G1 phase and a concomitant increase in the S phase fraction in PW‐treated nerves, closely mirroring the profile observed in the sham group (Figure 3D). These findings collectively indicate that PW enhances Schwann cell proliferative activity in vivo. Schwann cell subclustering resolved six distinct states, among which the Mki67^+^ subset, enriched in the PW group (13.5% vs. 4.5% in controls), exhibited high expression of repair‐associated genes such as *Birc5*, *Ube2c*, *Top2a*, and *Cdk1* (Figure 3E,F; Figure S9A–C). Intriguingly, direct exposure of Schwann cells to PW in vitro failed to stimulate proliferation, implying an indirect mechanism orchestrated via intercellular crosstalk (Figure 3G).

**FIGURE 4:**
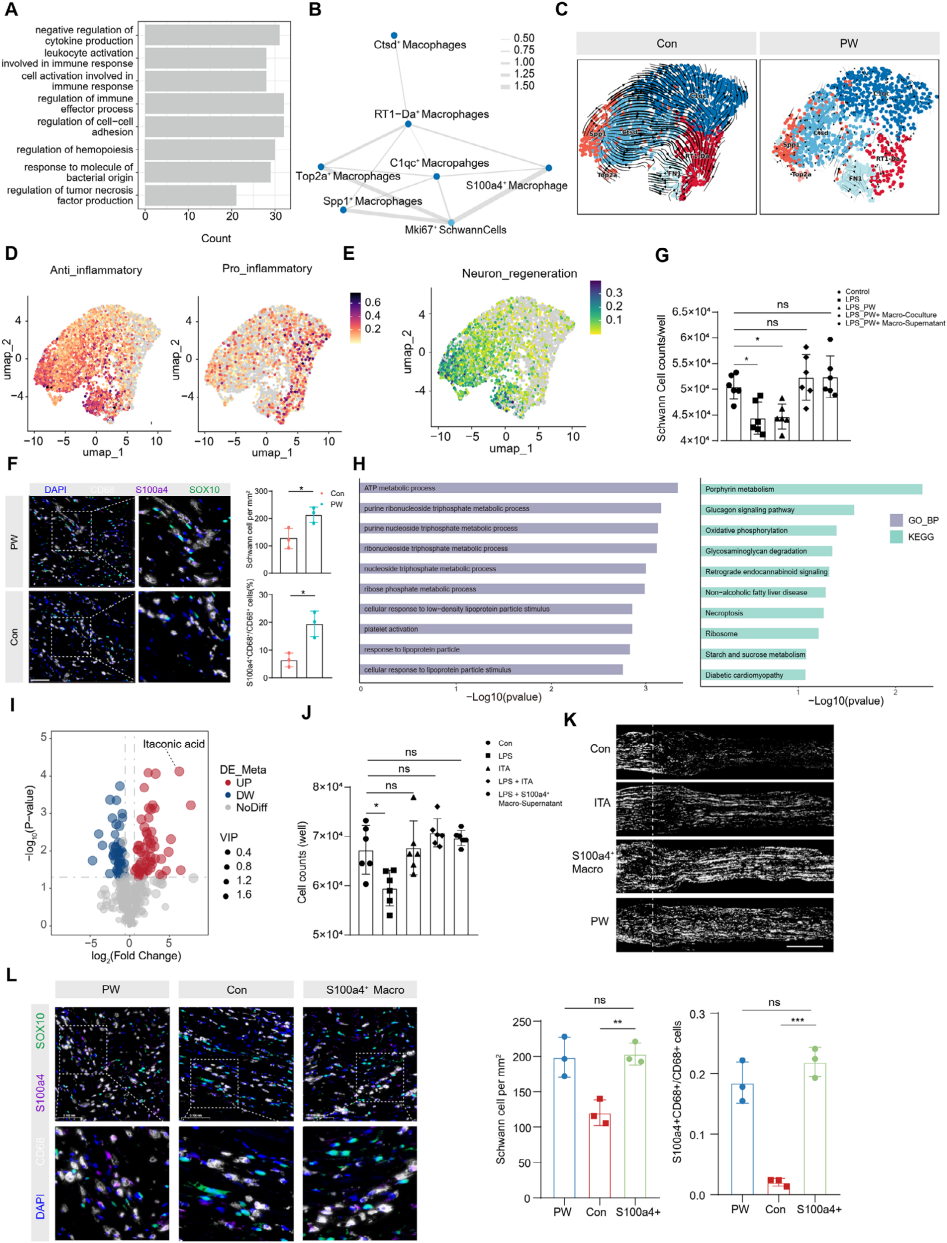
S100a4^+^ macrophage‐derived itaconate (ITA) promotes Schwann cell proliferation under inflammatory stress. (A) GO enrichment analysis of biological processes in RT1‐Da^+^ macrophages. (B) Interaction network between Mki67^+^ Schwann cells and macrophage subsets. (C) Pseudotime trajectory analysis of macrophage differentiation. (D,E) UMAP visualization of anti‐inflammatory (D) and pro‐inflammatory (E) macrophage states and neuronal repair signatures. (F) Multiplex immunohistochemistry (mIHC) and quantification of SOX10^+^ Schwann cells and S100a4^+^CD68^+^ macrophages (*n* = 3 /group). Scale bar: 100 µm. (G) Quantification of Schwann cell density across different conditions by Celigo Image Cytometer (*n* = 6/group). (H) GO and KEGG pathway enrichment analysis of macrophage transcriptomes. (I) Volcano plot of differentially expressed metabolites in macrophage supernatants. (J) Quantification of Schwann cell density under different conditions by Celigo Image Cytometer (*n* = 6/group). (K) Representative SCG‐10 immunostaining of sciatic nerve sections (*n* = 3/group). Scale bar: 500 µm. (L) mIHC analysis of Schwann cells and S100a4^+^ macrophages after adoptive transfer (*n* = 3/group). Green indicates SOX10, purple or white represents S100a4 or CD68, and blue indicates nuclei (DAPI). Scale bar: 100 µm. Mean values are shown, and error bars represent ± s.d., as analyzed by one‐way ANOVA with Tukey's post hoc tests in (G), (J) and (L) or unpaired Student's t test in (F). ns, not significant, ^*^ *p* < 0.05, ^**^ *p* < 0.01, ^***^ *p* < 0.001.

Cell–cell interaction analysis revealed that Schwann cells engage most robustly with macrophages (Figure 3H,I). Given the observed reduction in macrophage proportion and the known role of Schwann cell‐derived CCL2 in macrophage recruitment under inflammatory conditions (Figure S9D), we hypothesized that PW modulates macrophage polarization to indirectly foster Schwann cell proliferation. Re‐clustering of macrophages uncovered a striking increase in the S100a4^+^ subset and a significant decrease in RT1‐Da^+^ (MHC‐II^+^) macrophages upon PW treatment (Figure 3J,K; Figure S10A,B). The S100a4^+^ subset expressed high levels of reparative markers such as S100a4 and Fn1, lacking fibroblast‐associated markers such as *Col1a1, Col3a1, Pdgfra* and *Pdgfrb*, whereas RT1‐Da^+^ macrophages were enriched in antigen presentation pathways and associated with pro‐inflammatory T cell activation, potentially exacerbating nerve damage (Figure 4A). In addition, to determine whether S100a4^+^ /Fn1^+^ macrophages exist beyond our injury model, we analyzed independent, previously published single‐cell datasets from dermatomyositis [27] and hepatocellular carcinoma [28], a chronic inflammation‐associated malignancy. Notably, S100a4^+^ macrophages were consistently identified across these datasets, indicating that this macrophage substate is not unique to SNI nor restricted to an acute inflammatory context (Figure S11). Pseudotime trajectory analysis confirmed that PW induces a distinct macrophage differentiation path, uncorrelated with resident macrophage signatures (Figure 4C; Figure S12A,B). Signature scoring affirmed that S100a4^+^ macrophages exhibit a strong anti‐inflammatory and pro‐regenerative profile, while RT1‐Da^+^ macrophages display a pro‐inflammatory signature (Figure 4D,E). This reparative shift was further validated by qRT‐PCR, where PW suppressed pro‐inflammatory cytokines and promoted anti‐inflammatory and repair markers in LPS‐stimulated BMDMs (Figure S13). Spatial validation via multiplex immunohistochemistry confirmed concurrent increases in SOX10^+^ Schwann cells and S100a4^+^ CD68^+^ macrophages in PW‐treated nerves (Figure 4F).

To decipher the macrophage‐derived factor responsible for Schwann cell proliferation, we established a co‐culture system. Under inflammatory stress, soluble factors secreted by PW‐primed macrophages restored Schwann cell proliferation, whereas direct PW exposure did not (Figure 4G). GO and KEGG analyses of macrophage transcriptomes indicated enrichment in mitochondrial respiration, oxidative phosphorylation, and ATP metabolic processes (Figure 4H). Untargeted metabolomics of macrophage supernatants identified 61 upregulated metabolites in the PW group, with ITA exhibiting the most pronounced increase (Figure 4I; Figure S14A,B). To quantitatively evaluate ITA induction, we measured ITA in conditioned media from LPS‐polarized inflammatory macrophages treated with PW using ELISA. PW treatment led to a significant increase of ITA levels after 24 h, with a clear time‐dependent rise compared to earlier time points (0–12 h), indicating that PW promotes sustained ITA accumulation in the inflammatory macrophage secretome (Figure S15). These results support ITA as a durable immunometabolic output downstream of PW exposure in pro‐inflammatory macrophages. Interestingly, we found that in the absence of LPS stimulation, PW did not significantly promote ITA production, even after 48 h of treatment.

Exogenous ITA supplementation effectively restored Schwann cell proliferation under inflammatory conditions, recapitulating the effect of S100a4^+^ macrophage‐conditioned medium (Figure 4J). To determine whether ITA can directly influence Schwann cells (RSC96 cells) independently of macrophage cytokine changes, we treated Schwann cells with ITA or PBS for 24 h and performed bulk RNA‐seq. Gene set enrichment analysis (GSEA) revealed that ITA treatment significantly suppressed multiple inflammation‐associated pathways in Schwann cells, including inflammasome‐related programs (e.g., NLRP1 inflammasome complex assembly), respiratory burst, macrophage inflammatory protein‐1α production, leukocyte migration involved in inflammatory response, and broader chronic inflammatory response/wound‐response gene sets (Figure S16). These results indicate that ITA can directly suppress inflammatory responses, which identifies ITA as a previously unrecognized neuro‐immune mediator linking macrophage metabolism to Schwann cell replication.

To validate the central role of S100a4^+^ macrophages in vivo, we adoptively transferred S100a4^+^ macrophages sorted from PW‐treated BMDMs into SNI rats. This transfer phenocopied PW treatment, significantly elevating Schwann cell proportions and enriching the reparative macrophage pool (Figure 4K,L). In contrast, free ITA administration showed only transient efficacy due to rapid systemic clearance (Figure 4K; Figure S17A,B), underscoring the necessity of nanoparticle‐enabled sustained delivery. Together, these findings delineate a novel intercellular signaling axis in which PW reprograms macrophages into an S100a4^+^ anti‐inflammatory phenotype, resulting in sustained ITA secretion that selectively promotes Schwann cell proliferation under inflammatory stress.

### 2.4 Metabolic Reprogramming and Epigenetic Activation by PW Drive S100a4^+^ Macrophage Polarization

**FIGURE 5:**
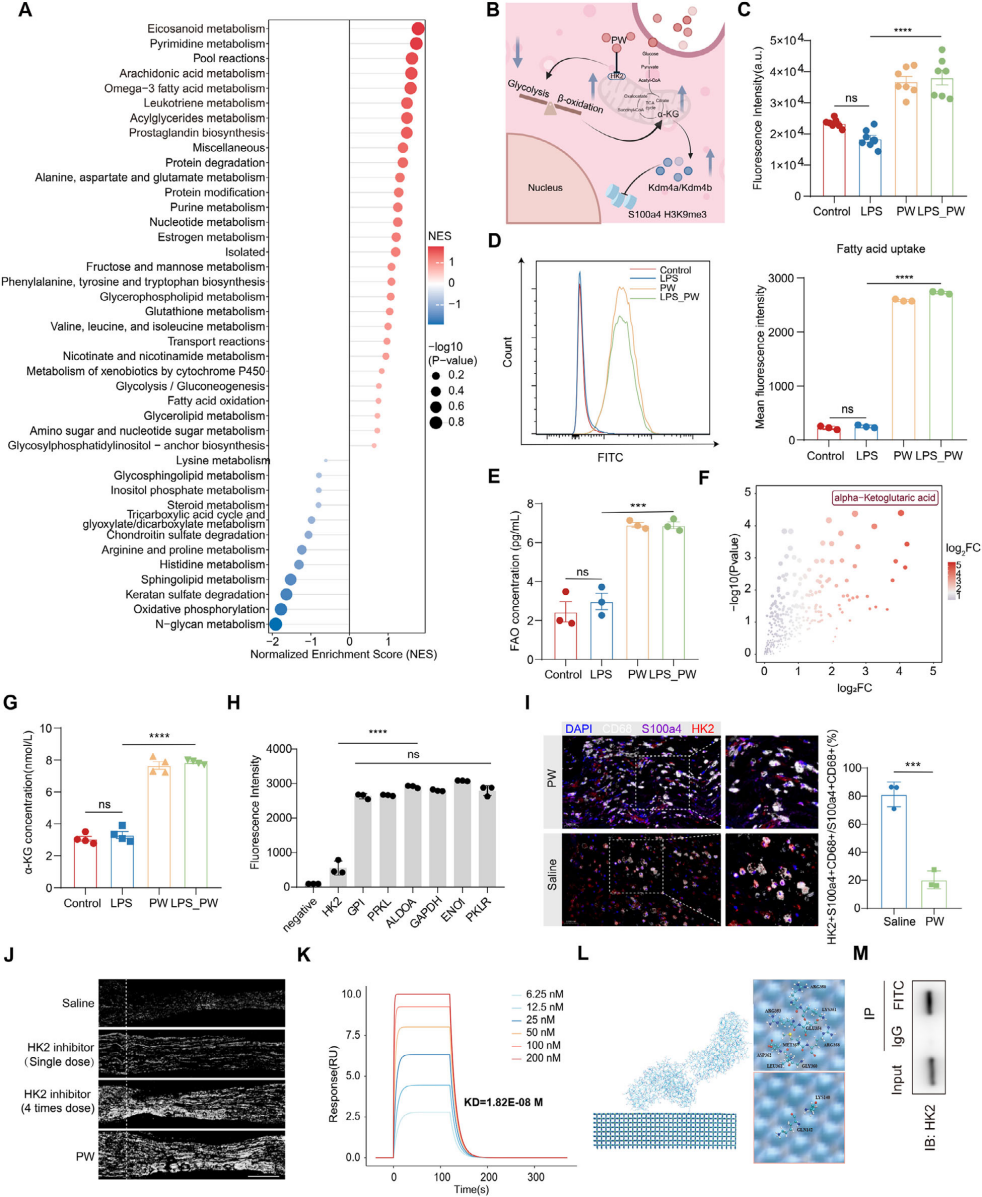
PW reprograms macrophage metabolism and epigenetics via HK2 targeting and α‐KG accumulation. (A) COMPASS analysis of metabolic alterations in macrophages after PW treatment. (B) Schematic of PW‐induced metabolic and epigenetic rewiring in macrophages. (C) Quantification of fatty acid uptake fluorescence intensity (*n* = 7/group). (D) Flow cytometry analysis of fatty acid uptake in BMDMs (*n* = 3/group). (E) Fatty acid β‐oxidation (FAO) levels in BMDMs (*n* = 3/group). (F) Scatter plot of metabolomic changes, highlighting α‐KG as the most significantly altered metabolite. (G) Quantification of α‐KG levels in BMDM supernatants (*n* = 4/group). (H) Flow analysis of glycolytic enzyme expression (*n* = 3/group). (I) mIHC of sciatic nerve sections showing HK2 expression and S100a4^+^ macrophage infiltration (*n* = 3/group). Scale bar: 100 µm. (J) SCG‐10 immunostaining of nerves after HK2 inhibitor or PW treatment (*n* = 3/group). Scale bar: 500 µm. (K) Surface plasmon resonance (SPR) analysis of PW‐HK2 binding affinity. (L) Molecular dynamics simulation of PW‐HK2 interaction. (M) Co‐immunoprecipitation of PW‐FITC and HK2 in LPS stimulated primary macrophages. Mean values are shown, and error bars represent ± s.d., as analyzed by one‐way ANOVA with Tukey's post hoc tests in (C–E) and (G–H), or t unpaired Student's *t* test in (I). ns, not significant, ^*^ *p* < 0.05, ^**^ *p* < 0.01, ^***^ *p* < 0.001, ^****^ *p* < 0.0001.

Having established the pivotal role of S100a4^+^ macrophages in orchestrating Schwann cell proliferation and nerve repair, we sought to elucidate the molecular mechanisms through which PW reprograms macrophage polarization. Given the intimate link between cellular metabolism and immune function, we first employed COMPASS analysis to systematically profile metabolic alterations in macrophages following PW treatment [29, 30]. Strikingly, among the top ten upregulated metabolites, six were associated with fatty acid metabolism (Figure 5A), pointing to a profound enhancement of fatty acid utilization pathways. Consistent with this, PW robustly promoted fatty acid uptake in BMDMs under both basal and LPS‐stimulated conditions (Figure 5B–D). Furthermore, fatty acid β‐oxidation (FAO), which serves as a central pathway in fatty acid catabolism [31], was significantly augmented upon PW treatment, as evidenced by ELISA‐based quantification (Figure 5E). Intriguingly, however, the intracellular level of acetyl‐CoA, the canonical end‐product of FAO, remained unchanged (Figure S18), suggesting a potential rerouting of metabolic flux.

To resolve this paradox and gain an unbiased view of PW‐induced metabolic shifts, we performed untargeted metabolomics on macrophage lysates. This analysis revealed a pronounced accumulation of α‐KG in PW‐treated macrophages under inflammatory challenge (Figure 5F), a finding corroborated by ELISA (Figure 5G). Since α‐KG serves as a critical TCA cycle intermediate and its pool can be replenished via acetyl‐CoA‐driven anaplerosis [32, 33], these results indicate that PW redirects metabolic flux from glycolysis toward FAO, culminating in elevated α‐KG levels. Our earlier Human Microarray Proteomic Analysis had identified glycolysis as a major pathway inhibited by PW (Figure 1E). Integrative analysis now pinpointed HK2, the gatekeeping enzyme of glycolysis, as a key target suppressed by PW in BMDMs (Figure 5H; Figure S19A–C). Spatial validation via multiplex immunohistochemistry (mIHC) of injured sciatic nerves confirmed that PW administration concurrently boosted the infiltration of S100a4^+^ macrophages and diminished HK2 expression within these cells (Figure 5I). To directly assess the therapeutic contribution of HK2 inhibition, we compared axonal regeneration following single or repeated doses of an HK2 inhibitor (under ultrasound guidance) with PW treatment. Notably, repeated HK2 inhibition or PW treatment significantly enhanced axonal regrowth (Figure 5J) only when administered repeatedly rather than as a single dose, underscoring the necessity of sustained glycolytic suppression for effective nerve repair.

We next probed the physical interaction between PW and HK2. Surface plasmon resonance (SPR) demonstrated high‐affinity binding with a dissociation constant (*K*D) of 1.82 × 10^−8^ m (Figure 5K). Molecular dynamics simulations further delineated that PW engages key residues (R350, R353, E354, M357, R358, D362, and Q142) within the N‐terminal domain of HK2 (residues 350–362), forming a stable complex (Figure 5L; Figure S20A). System stability was confirmed by root‐mean‐square deviation (RMSD) analysis during the 90–100 ns simulation window (Figure S20B). To validate the intracellular interaction between PW and HK2, co‐immunoprecipitation assay was performed in primary rat macrophages incubated with PW–FITC. Immunoblot analysis revealed a clear enrichment of HK2 in the anti‐FITC immunoprecipitated fraction, whereas no detectable HK2 signal was observed in the IgG control (Figure 5M). In addition, multicolor immunofluorescence analysis was performed in primary rat macrophages, with PW was fluorescently labeled via FITC conjugation. Confocal imaging demonstrated that PW‐FITC showed prominent intracellular co‐localization with HK2, whereas free FITC exhibited diffuse and quenched signals, predominantly remaining extracellular (Figure S21). These data support HK2 as a bona fide intracellular target of PW in macrophages. In addition, pro‐inflammatory macrophages were treated with PBS, PW, or an HK2 inhibitor for 24 h, followed by bulk RNA sequencing analysis. Genome‐wide correlation analysis according to previous report [34] revealed a strong concordance between PW‐treated and HK2 inhibitor‐treated macrophages (Pearson r = 0.963, p<0.0001, Figure S22), indicating that PW largely recapitulates the transcriptional program induced by HK2 inhibition and the functional link between PW's binding to HK2.

**FIGURE 6:**
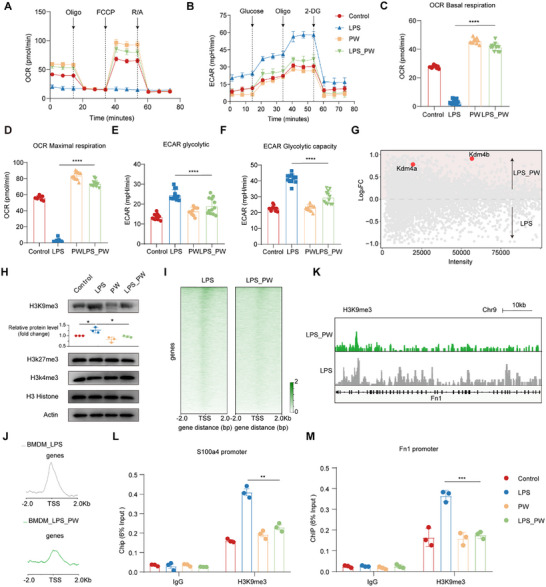
PW reverses LPS‐induced mitochondrial suppression and facilitates H3K9me3 demethylation at regenerative gene loci. (A,B) Oxygen consumption rate (OCR) and extracellular acidification rate (ECAR) profiles of BMDMs under different treatments (Control, *n* = 9; LPS, *n* = 11; PW, *n* = 11; LPS_PW, *n* = 11). (C,D) Basal and maximal respiration levels derived from OCR data (Control, *n* = 9; LPS, *n* = 11; PW, *n* = 11; LPS_PW, *n* = 11). (E,F) Glycolytic flux and capacity derived from ECAR data. (G) Scatter plot of epigenetic regulators, highlighting upregulation of Kdm4a and Kdm4b. (H) Western blot analysis of histone modifications (H3K9me3, H3K27me3, H3K4me3) (*n* = 3/group). Actin served as a loading control. (I) Heatmap of H3K9me3 occupancy across the genome. (J) Average H3K9me3 peak intensity in BMDMs. (K) CUT&Tag sequencing tracks of H3K9me3 at the S100a4 gene locus. (L,M) ChIP‐qPCR analysis of H3K9me3 enrichment at S100a4 and Fn1 promoter regions (*n* = 3/group). Mean values are shown, and error bars represent ± s.d., as analyzed by one‐way ANOVA with Tukey's post hoc tests in (C–F), (H) and (L–M). ns, not significant, ^*^ *p* < 0.05, ^**^ *p* < 0.01, ^***^ *p* < 0.001, ^****^ *p* <0.0001.

Functionally, PW impeded glucose uptake in inflammatory BMDMs (Figure S23A,B), and extracellular flux analysis revealed that PW treatment reversed LPS‐induced mitochondrial respiratory suppression while attenuating aerobic glycolysis, as indicated by increased oxygen consumption rate (OCR) and decreased extracellular acidification rate (ECAR) (Figure 6A–F). Collectively, these data establish that PW directly targets HK2 to suppress glycolysis, thereby shunting metabolic flux toward mitochondrial oxidation and elevating intracellular α‐KG. Given the well‐established role of α‐KG as an essential cofactor for Jumonji C‐domain‐containing histone demethylases, we hypothesized that PW‐induced α‐KG accumulation might instigate epigenetic reprogramming. Untargeted proteomics identified significant upregulation of Kdm4a and Kdm4b (Figure 6G), demethylases that specifically catalyze the removal of the repressive histone mark H3K9me3 [35, 36, 37]. While global histone H3 levels and other modifications (H3K27me3, H3K4me3) were unaffected, LPS stimulation markedly increased H3K9me3 levels, which were substantially reversed by PW treatment (Figure 6H).

We then employed Cleavage Under Targets and Tagmentation (CUT&Tag) sequencing to map genome‐wide H3K9me3 occupancy. Macrophages treated with LPS_PW exhibited a global reduction in H3K9me3 binding compared to LPS‐only controls (Figure 6I,J). Notably, this erasure was particularly evident at the *S100a4* promoter and exon regions (Figure 6K). Chromatin immunoprecipitation quantitative PCR (ChIP‐qPCR) confirmed that LPS‐induced H3K9me3 enrichment at the *S100a4* and *Fn1* loci was robustly alleviated by PW, concomitant with restored gene expression (Figure 6L,M). These findings mechanistically link PW‐induced metabolic rewiring to epigenetic activation of a pro‐regenerative macrophage phenotype.

### 2.5 Translational Validation: Functional Restoration in a Canine Model

**FIGURE 7:**
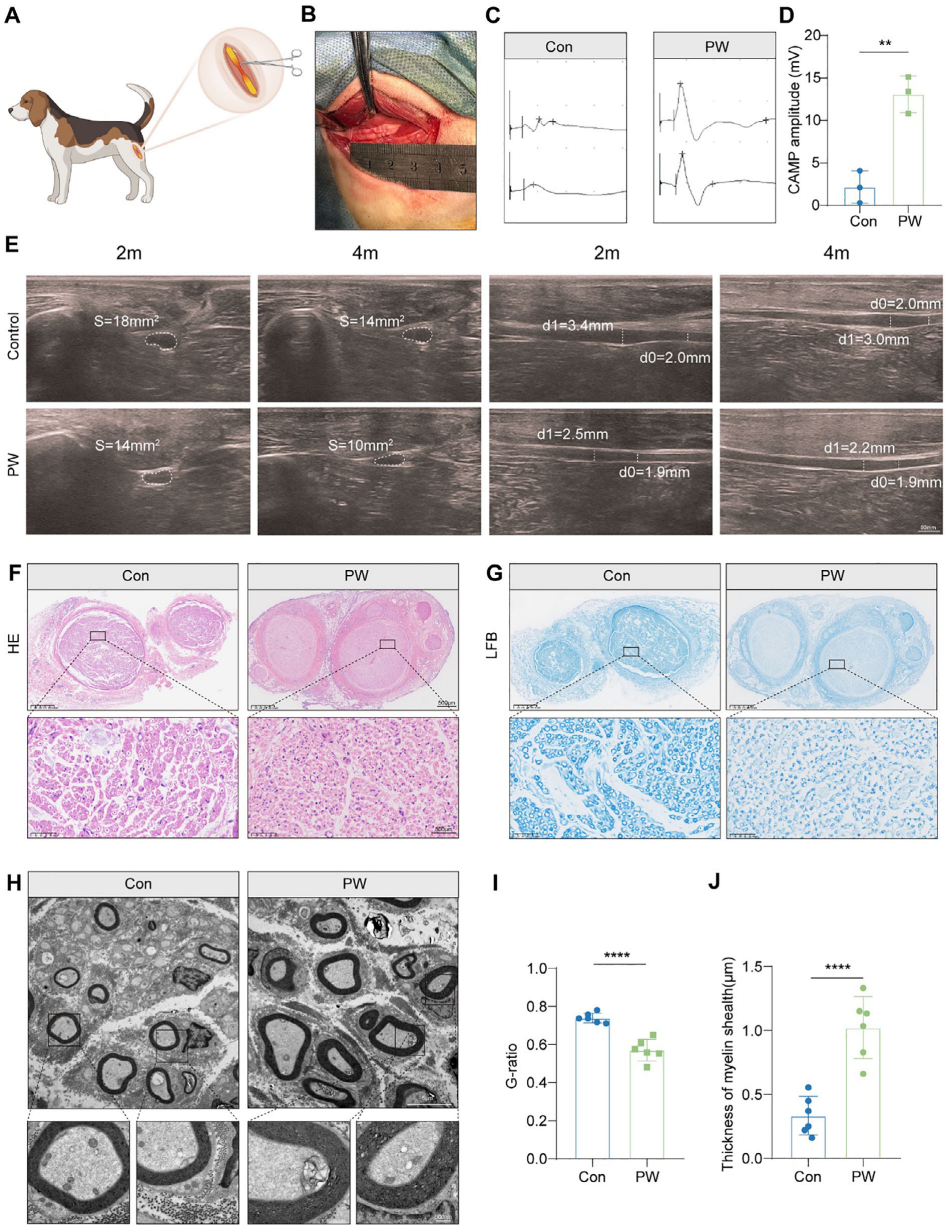
Therapeutic efficacy of PW in a canine model of sciatic nerve injury. (A) Schematic of the canine SNI model and PW treatment regimen. (B) Intraoperative image showing sciatic nerve exposure and PW administration. (C) Representative CMAP recordings from control and PW‐treated groups at 4 months. (D) Quantification of CMAP amplitudes (*n* = 3/group). (E) Ultrasonographic images and measurements of nerve cross‐sectional area (S) and diameter (d) at 2 and 4 months. (F) H&E staining showing improved nerve architecture and reduced vacuolation in the PW group. (G) Luxol Fast Blue (LFB) staining indicating enhanced myelination. (H) TEM images of myelinated axons; insets show detailed myelin structure. Scale bars: 5 µm (main), 500 nm (insets). (I,J) Quantitative analysis of g‐ratio and myelin sheath thickness. *n* = 3 biologically independent animals per group. For each biological sample, two regions of interest were randomly selected to ensure unbiased data representation. Mean values are shown, and error bars represent ± s.d., as analyzed by one‐way ANOVA with t unpaired Student's *t* test in (D), (I–J). ^**^ *p* < 0.01, ^****^ *p* < 0.0001.

Building upon the compelling evidence from rodent studies, we sought to validate the therapeutic efficacy and translational potential of PW in a clinically relevant canine SNI model, a critical step toward bridging the gap between preclinical innovation and clinical application. The canine model not only recapitulates key anatomical and physiological features of human peripheral nerves but also provides a more rigorous assessment of functional recovery and long‐term biocompatibility. To this end, we evaluated the systemic safety and regenerative outcomes of PW over a 4‐month postoperative period (Figure 7A,B). Systemic biosafety is a prerequisite for clinical translation. Comprehensive serum biochemical analyses at 4 months post‐treatment revealed no significant alterations in markers of liver function (AST, ALT), renal function (BUN), or general tissue damage (LDH) in PW‐treated animals compared to controls (Figure S24A–D). These findings underscore the favorable biocompatibility and minimal off‐target effects of PW, even under prolonged exposure in a large mammal.

Functional recovery was rigorously assessed via electrophysiological measurements. Notably, CMAP amplitudes, a direct correlate of neuromuscular integrity and axonal conductivity, were significantly higher in the PW group relative to controls at the 4‐month endpoint (Figure 7C,D). This electrophysiological improvement reflects not only enhanced reinnervation but also the restoration of synaptic transmission, underscoring the functional relevance of PW‐induced regeneration. Non‐invasive ultrasonography provided dynamic and quantitative insights into structural recovery. PW‐treated nerves exhibited a progressive reduction in cross‐sectional area and diameter over the 2‐ and 4‐month intervals, indicative of diminished edema, improved axonal compaction, and accelerated tissue remodeling (Figure 7E). These imaging findings align with electrophysiological data, collectively supporting the notion that PW facilitates both morphological and functional restoration. Histopathological evaluations further corroborated these observations. H&E staining of PW‐treated nerves revealed superior axonal alignment, reduced vacuolation, and diminished fibrotic scarring compared to controls (Figure 7F). LFB staining demonstrated more uniform and thicker myelin sheaths, consistent with enhanced remyelination and Schwann cell‐mediated repair (Figure 7G). Ultrastructural analysis via transmission electron microscopy (TEM) provided the highest‐resolution validation: PW‐treated specimens exhibited a significant increase in myelin sheath thickness and a corresponding decrease in g‐ratio, both hallmarks of mature, functional myelination (Figure 7H–J). These structural improvements are consistent with the previously elucidated mechanism wherein PW promotes Schwann cell proliferation and macrophage‐mediated support via ITA secretion and epigenetic reprogramming.

## 3 Discussion

Our findings elucidate a coordinated metabolic‐epigenetic axis wherein PW, through high‐affinity binding to HK2, inhibits glycolytic flux while enhancing fatty acid oxidation, consequently resulting in the accumulation of α‐KG (Figure 5). This metabolic shift serves as a substrate boost for Kdm4a/b‑dependent demethylation of the repressive histone mark H3K9me3, thereby relieving transcriptional repression at key regenerative loci such as S100a4 and promoting macrophage polarization toward a pro‑regenerative phenotype (Figure 6). To contextualize the novelty of this work, it is important to consider recent advances in nanomaterial‐enabled nerve repair. Current strategies have largely adhered to a “single‐target, delivery‐centric” paradigm [38, 39]. In this paradigm, nanomaterials primarily function as sophisticated carriers for exogenous agents including anti‐inflammatory drugs, growth factors, genetic materials, or gasotransmitter donors, aimed at transiently suppressing inflammation, mitigating oxidative stress, or providing neurotrophic support [40, 41, 42, 43, 44]. While these approaches have made significant strides in fostering a more permissive regenerative environment, they often focus on isolated pathological nodes. This may not adequately address the highly interconnected and dynamic injury microenvironment, where dysfunctional immune‑glial crosstalk is governed by intertwined metabolic and epigenetic networks [45]. Moreover, the transient action of released cargos and the rapid systemic clearance of free molecular agents (e.g., HK2 inhibitors or itaconate; Figure S17) can limit the sustained cellular reprogramming essential for complete regeneration.

Our study introduces a paradigm shift through PW, which functions not as a passive carrier but as an intrinsically bioactive nanomodulator that enables “material‑mediated cell reprogramming.” This approach is distinguished by several integrated features: (1) Sustained microenvironmental engagement: PW exhibits prolonged in situ retention (Figure 1G), delivering persistent immunometabolic modulation that overcomes the rapid pharmacokinetics of free molecular agents (e.g., HK2 inhibitors or itaconate; Figure S17). (2) Direct targeting of a core metabolic node: PW acts as a high‑affinity ligand for HK2, directly inhibiting glycolysis at its source and initiating metabolic rewiring without relying on encapsulated drugs (Figure 5K,L). (3) Coupled metabolic‑epigenetic reprogramming: The resulting metabolic shift elevates intracellular α‑KG, which in turn fuels Kdm4a/b‑mediated H3K9me3 demethylation at pro‑regenerative gene promoters, establishing a direct causal link from nanoparticle‑induced metabolic perturbation to epigenetic activation (Figure 6). (4) Orchestration of a self‑amplifying therapeutic cascade: This reprogramming drives macrophages into a novel S100a4^+^ pro‑regenerative substate, which secretes itaconate to directly support Schwann cell proliferation under inflammatory stress (Figure 4). Thus, PW coordinates a holistic cascade encompassing metabolic inhibition, epigenetic activation, and restorative paracrine signaling that actively restores the essential macrophage‐Schwann cell dialogue.

By integrating these multi‑level regulatory functions into a single nanomaterial entity, our strategy advances beyond the sequential application of discrete therapies. It represents a systems‑intervention approach in which the nanomaterial is designed to engage and rewire the intrinsic regulatory circuits of target cells. The robust functional recovery demonstrated in both rodent and canine models underscores the translational potential of this paradigm. Beyond peripheral nerve repair, the “material‑mediated cell reprogramming” concept provides a versatile framework that could be adapted to treat a range of neurodegenerative and inflammatory conditions characterized by dysfunctional immunometabolic‑epigenetic crosstalk. This work establishes a nanomaterials‐based strategy for immunometabolic‐epigenetic remodeling that restores macrophage‐Schwann cell communication and promotes nerve regeneration. This approach not only provides insights into the metabolic and epigenetic regulation of nerve repair but also offers a versatile platform with broad implications for the treatment of neurodegenerative diseases.

In summary, this study presents the development and mechanistic elucidation of a biomimetic PW as a potent and durable nanomodulator, exemplifying a ‘material‐mediated cell reprogramming’ strategy for sciatic nerve regeneration. Moving beyond conventional drug delivery paradigms, PW exhibits intrinsic bioactivity that enables sustained local retention and direct targeting of key metabolic pathways, representing a significant advantage over rapidly cleared molecular agents. The core mechanistic breakthrough lies in the elucidation of a coherent, nanomaterials‐enabled axis that integrates immunometabolic reprogramming with epigenetic regulation. We demonstrate that PW, via high‐affinity binding to HK 2, shunts macrophage metabolism from glycolysis toward fatty acid oxidation, resulting in the accumulation of α‐KG. This metabolic shift fuels Kdm4a/b‐dependent demethylation of the repressive histone mark H3K9me3 at loci of regenerative genes, including *S100a4*, thereby driving macrophage polarization toward a previously unrecognized pro‐regenerative substate. Furthermore, we identify ITA as a crucial neuro‐immune mediator secreted by these reprogrammed macrophages, which directly support Schwann cell proliferation under inflammatory stress.

Collectively, this study provides a generalizable paradigm for “material‐mediated cell reprogramming,” where a synthetic nanomaterial precisely orchestrates intercellular crosstalk through coordinated metabolic and epigenetic mechanisms. The robust efficacy validated in both rodent and canine models underscores the strong translational potential of this approach. By bridging the fields of nanomaterial science, immunometabolism, and epigenetics, our work not only offers a promising therapeutic strategy for peripheral nerve repair but also establishes a versatile platform that could be adapted to treat a broad spectrum of neurodegenerative and inflammatory diseases characterized by dysfunctional immunometabolic‐epigenetic communication.

## 4 Experimental Section/Methods

### 4.1 Synthesis of PW

To synthesize PW, manganese chloride (10 mg) was dissolved in 10 mL of BSA solution (100 mg) to prepare Solution A. Similarly, potassium ferrocyanide (17.8 mg) was dissolved in 10 mL of BSA solution (100 mg) to prepare Solution B. The two solutions were gradually combined under continuous magnetic stirring for 2 h, resulting in a white colloidal solution. The mixture was then incubated at 4°C for 14 h to allow complete reaction. HW particles were harvested by centrifugation at 15,000 rpm for 15 min, followed by thorough washing with deionized water to remove residual reactants.

### 4.2 Cell Culture

The RSC96 Schwann cell line (GNR6) and the rat pheochromocytoma cell line PC12 (SCSP‐517) were obtained from the Type Culture Collection of the Chinese Academy of Sciences. Primary BMDMs were isolated from the femurs and tibias of 8‐ to 12‐week‐old Sprague‐Dawley (SD) rats. After lysis of erythrocytes, bone marrow cells were cultured at 37°C in 5% CO_2_ in BMDM medium, which consisted of Dulbecco's modified Eagle medium (DMEM; GIBCO) supplemented with 10% fetal calf serum (Biochrom), 1% penicillin‐streptomycin (GIBCO), and 20 ng mL^−1^ recombinant rat M‐CSF (Thermo Fisher). Non‐adherent cells were removed, and the remaining cells were replated in fresh BMDM medium. Cultures were maintained for 5 days to allow macrophage differentiation.

### 4.3 Human Microarray Proteomic Analysis

Protein binding microarray chips comprised of ≈20000 individual human GST (glutathione S‐transferase)‐ and His‐tagged full‐length proteins were obtained from the Johns Hopkins Medical Institutions Protein Microarray Core (CDI Laboratories, Inc).The microarray proteome analysis was performed according to the procedure detailed below, and the experiments and data processing were performed by Wayen Biotechnology Company (Shanghai, China).

To assess preferential protein associations of PW, PW was biotinylated (PW–biotin) and incubated with HuProt microarrays, while biotin alone was used as a parallel negative control under identical conditions. HuProt proteome microarrays were blocked with blocking buffer (5% BSA [bovine serum albumin] in 1×TBST [Tris‐Buffered Saline with Tween 20], pH = 7.5) for 1.5 h at room temperature, then washed with 1× TBST for 5 min.

PW–biotin or biotin‐only control (10 µmol/L) was then incubated with the blocked microarrays for 1 h at room temperature. Thereafter, the microarrays were washed 3× with TBST, for 5 min each time, and Cy5‐Streptavidin was added (1:1000 dilution). Following incubation with Cy5‐Streptavidin, the microarrays were again washed with TBST (3×, 5 min each time). Finally, they were spun dry for 2 min, then scanned with an Axon GenePix 4000B. The GenePix Pro 6.0 software (Axon Instruments) was used to extract data from the recorded microarray images. Protein spots were identified based on differential Z‐score analysis by comparing PW–biotin–treated microarrays with biotin‐only controls, rather than absolute signal intensity. The Z‐Score calculation formula is as follows.

$$M=\mathrm{Median}\;\left(I_{x,y,z}\right)$$

$$Z-\mathrm{Scor}e_{x,y,z}=\left(I_{x,y,z-}M\right)/\mathrm{SD}$$

M: median of all protein sites Ix,y,z; Ix,y,z: raw signal intensity of each protein site in the chip; x: column number of each protein site; y: each protein The row number of the site; z: the sequence number of the block on the chip. Z‐Scorex, y, z: Z‐Score value of each protein site within the chip; SD: standard deviation (SD) of all Ix, y, z.

The enrichment analysis, including the Kyoto Encyclopedia of Genes and Genomes. (KEGG) pathway and Gene Ontology was performed using the clusterProfiler package in RStudio.

### 4.4 Bioluminescence Imaging

To enable fluorescence visualization, amine‐functionalized Cy5.5 was conjugated to PW, BSA, and ITA, resulting in Cy5.5‐PW, Cy5.5‐BSA, and Cy5.5‐ITA, respectively. The conjugates were purified via centrifugation at 15 000 rpm for 5 min to remove any unbound Cy5.5. For in vivo application, 30 µL of the prepared suspension was injected into the perineural tissue surrounding the SNI site in Sprague–Dawley (SD) rats (n  = 3 biologically independent animals per group). Injections were performed under sterile conditions using a Hamilton microsyringe (Hamilton, USA) to ensure precise localization near the injury. Fluorescence imaging was conducted using the VISQUE InVivo imaging system to monitor the distribution and retention of Cy5.5‐PW at 1, 3, 7, and 14 days post‐injection. Fluorescence intensity at the injection site was quantified using Living Image software (BIOTIMES, China).

### 4.5 The Construction of SNI Surgery Models

All rodent experimental procedures were approved by the Animal Welfare Ethics Committee of Shanghai Jiao Tong University School of Medicine Affiliated Sixth People's Hospital (No. DWLL2025‐0006). Adult male Sprague–Dawley (SD) rats (250–300 g, 8–10 weeks old) were used for this study (*n*  = 10 biologically independent animals per group). Sample sizes for each experiment are detailed in the corresponding figure legends. Rodents were anesthetized via intraperitoneal injection of pentobarbital sodium (0.05 mg/g body weight). The hind limbs and lower back were shaved and sterilized with iodine, and ophthalmic solution was applied to the eyes to prevent drying. A skin incision was made, and the biceps femoris and gluteus superficialis muscles were separated using blunt dissection to expose the sciatic nerve with a surgical hook. SNI was performed approximately 20 mm distal to the sciatic dorsal root ganglion (DRG) by compressing a 3‐mm nerve segment with #5 forceps three times for 15 s each. The crush site was marked with India ink. Subsequently, 30 µL of PW or saline were injected around the injury site using a microsyringe (Hamilton, USA). After the procedure, the muscles and skin were sutured, and the animals were allowed to recover in a warm environment. Amoxicillin was provided in drinking water for one‐week post‐surgery to prevent infection.

### 4.6 CatWalk Gait Analysis

To assess functional recovery at 2 and 4 weeks post‐surgery, rats underwent gait analysis using the CatWalk XT 10.6 system (Noldus Information Technology, Wageningen, The Netherlands). The system consists of an illuminated glass walkway and a high‐speed camera positioned below the walkway to capture paw prints as the animals traverse the platform. Prior to baseline recording, rats were acclimated to the system for three consecutive days. Each habituation session involved allowing the animals to explore the walkway freely, without external stimuli, to ensure they became familiar with the environment. During each session, rats were placed at the starting platform and permitted to cross the walkway spontaneously. Only uninterrupted crossings—defined as those with no pausing, rearing, or turning back—were included in the analysis. A minimum of three valid crossings per animal was recorded to ensure consistency and reproducibility. The walkway illumination intensity was set at [illumination intensity], and camera sensitivity was adjusted to optimally capture paw placements, minimizing background noise. Data acquisition was conducted using the default settings of the CatWalk XT system, with a minimum intensity threshold of [value]. The recorded data was analyzed using the CatWalk XT software.

### 4.7 Sensory Function Test

Thermal perception was assessed using the hot plate test at 2 and 4 weeks post‐surgery, as previously described. Testing was conducted in a quiet environment, with the temperature of the plate set to 54°C. The animals' response latency was measured, with a cutoff time of 30 s to avoid injury. Prior to baseline testing, rats were habituated to the setup for three consecutive days to minimize stress and ensure familiarity with the procedure. During testing, each rat was placed on the heated plate, and the time taken for the animal to exhibit a thermal response, such as paw licking or paw lifting, was recorded. A maximum of 30 s was allowed before the animal was removed from the plate to prevent tissue damage.

### 4.8 Histological Analyze

Regenerative nerve tissue and muscle samples from rats and beagles were subjected to H&E and LFB staining for histological analysis. Nerve samples were fixed in 4% paraformaldehyde (PFA) overnight, followed by a dehydration process. These samples were then embedded and sectioned into 10 µm‐thick cross‐sections using an acryostat. The sections were stained with H&E or 1% LFB, and images were captured using an optical microscope. For muscle analysis, samples from the gastrocnemius muscle on the injured side were processed similarly. Paraffin‐embedded tissue was sectioned into 7‐µm‐thick slices and stained with H&E and Masson's trichrome stain (Sinopharm) to evaluate muscle pathology and fibrosis.

### 4.9 Sciatic Nerve Regeneration

At 14 or 28 days following surgical intervention, the sciatic nerves were meticulously dissected and subjected to post‐fixation in 4% paraformaldehyde (PFA) for a standardized duration of 1 h at 4°C. The tissue specimens were subsequently transferred to a 30% sucrose solution and maintained for a minimum period of 3 days to ensure optimal cryoprotection. Thereafter, the tissues were embedded in Tissue‐Tek Optimal Cutting Temperature (OCT) compound and cryopreserved at −80°C until the sectioning procedure. Serial sagittal sections of the frozen tissue were prepared for subsequent histological analysis. Immunohistochemical staining was conducted employing an anti‐SCG‐10 antibody (1:800 dilution, rabbit polyclonal, Novus Biologicals) to specifically label regenerating axonal structures. The precise localization of the nerve crush site was determined through morphological identification of nerve deformation and axonal disruption, which corresponded anatomically with the region exhibiting the highest intensity of SCG‐10 immunoreactivity.

### 4.10 Immunohistochemistry

Immunohistochemistry was performed on tissue sections following standard procedures. The sections were rehydrated with PBS and then blocked and permeabilized for 1 h using either 8% BSA (Sigma Aldrich) or 10% normal goat serum (Abcam) containing 0.3% PBS‐Triton X‐100. Subsequently, the sections were incubated overnight at room temperature with primary antibodies: anti‐SCG‐10 (1:800, Novus), anti‐SOX10 (1:1000, Abcam), anti‐CD68 (1:1000, Abcam), anti‐S100β (1:1000, Abcam), anti‐NF200 (1:1000, Abcam), anti‐βIII Tubulin (1:3000, Abcam), anti‐S100a4 (1:2000, Abcam), and anti‐HK2 (1:2000, Abcam). After incubation, the sections were washed three times with PBS and then incubated with Alexa Fluor‐conjugated secondary antibodies for 1 h. Slides were scanned using the PerkinElmer Vectra3 platform, and images were quantified using Inform software (V.2.6.0).

### 4.11 Electrophysiology Assessmen

To evaluate the electrophysiological properties of rats at 4 weeks post‐surgery and beagles at 4 months post‐surgery, the sciatic nerves of the experimental side were re‐exposed under general anesthesia. Bipolar hooked stimulating electrodes were placed on the sciatic nerve trunk at the proximal end, and CMAP were recorded from the gastrocnemius muscle on the ipsilateral side using an 8‐channel physiological signal recorder (RM‐6280C). Each experiment was repeated three times per group to ensure consistency and reliability of the results.

### 4.12 TEM Detection

Transmission electron microscopy (TEM; Olympus, Tokyo, Japan) was employed to evaluate nerve regeneration at 28 days post‐surgery. Nerve specimens were initially fixed in 2% PBS‐buffered osmium tetroxide and glutaraldehyde for 2 h at 4°C. The specimens were then dehydrated through a graded ethanol series before being infiltrated with epoxy resin and embedded transversely. Ultrathin sections (50 nm) were prepared using an ultramicrotome (Leica, Wetzlar, Germany). These sections were placed on copper grids and stained with lead citrate to prepare them for TEM analysis.

In the TEM images, the evaluation of the myelin sheath thickness and the g‐ratio (defined as the inner neurite diameter divided by the outer diameter of the myelinated sheath) was carried out by random fields of each TEM image.

### 4.13 Ultrasonic Real‐Time Imaging

Ultrasound imaging was employed to dynamically monitor sciatic nerve recovery at various time points. For rats, imaging was performed on the day before surgery (baseline), and at 1, 2, 3, and 4 weeks post‐surgery. For Beagle dogs, imaging was conducted on the day before surgery and at 2 and 4 months post‐surgery. For rats, anesthesia was administered prior to imaging, and the hindlimbs were shaved to optimize imaging conditions. The animals were positioned in a prone posture with their hip joints extended and limbs fixed to ensure stability during the procedure. Ultrasound imaging was conducted using a [device model] with a depth setting of 1.5 cm, gain set to 50%, and a frequency of 22 MHz. Adequate ultrasound coupling gel was applied between the probe and the muscle to ensure optimal contact and signal transmission.

The ultrasound probe was placed transversely on the mid‐posterior thigh, medial to the femur, at an approximate angle of 75° relative to the horizontal plane. The sciatic nerve was identified beneath the biceps femoris muscle and adjacent to the medial aspect of the femur. The nerve was visualized and tracked in both transverse and longitudinal planes along its entire course in the posterior thigh. Tracking proceeded upward to the level of the greater trochanter and downward to the bifurcation of the nerve into the tibial, sural, and common peroneal branches. The bifurcation site was documented using femoral bony landmarks and surrounding musculature for reference. For Beagle dogs, the same methodology was applied, with the exception of anesthesia.

### 4.14 scRNA‐Seq Analysis

Nerve samples from rats were processed immediately after dissection to generate single‐cell suspensions (*n* = 3 biologically independent animals per group). The raw scRNA‐seq data from spontaneous tumors were processed using CellRanger v6.1.2 (10x Genomics), with reads aligned to the mouse GRCm38 reference genome to generate a UMI count matrix. Cells were filtered based on the following criteria: total UMI count exceeding 40 000, detection of fewer than 300 or more than 7000 genes, and mitochondrial gene content exceeding 15%. Cells that did not meet these criteria were excluded from further analysis. To estimate and remove potential doublets, the DoubletFinder R package (v2.0.3) was used, assuming a 5% doublet rate.

Pre‐processing followed standard Seurat v4.3.0.1 pipelines. Data normalization was performed using the NormalizeData function with a scale factor of 10 000. The 2000 most variable genes were selected using the FindVariableFeatures function. Gene expression was then scaled by regressing out mitochondrial gene percentages, total UMI counts, and detected features. The processed data were subjected to principal component analysis (PCA), and the top 20 principal components were used for Uniform Manifold Approximation and Projection (UMAP), resulting in a 2D reduction of the dataset. Nearest neighbor graphs were constructed using the FindNeighbors function, and cell clusters were identified with the FindClusters function at a resolution of 0.5. The major cell types were annotated based on the expression of known marker genes: T and NK cells (Cd3e, Cd8a, Nkg7, Gzmb), cDC cells (Cd83, Ccl22, Cd11c), pDC cells (Siglech, Irf8, Bst2, Ly6d), Schwann cells (Mbp, Pmp22, Ngrf, Cxcl14), B cells (Cd79a, Cd79b, Ighm), macrophages (Cd68, Adgre1, Cq1a, Itgam), mural cells (Acta2, Myh11, Tagln, Pdgfrb), neutrophils (S100a8, S100a9, Ly6g, Cxcr2), fibroblasts (Postn, Pdgfa, Dpt, Lum), and endothelial cells (Eng, Vwf, Ephb4, Cldn5). Signature scores were calculated based on the AddModuleScore function in the Seurat R package.

For cross‐context validation, publicly available single‐cell RNA‐seq datasets from dermatomyositis [27] and hepatocellular carcinoma [28], a chronic inflammation‐associated malignancy, were reanalyzed using consistent annotation strategies. The presence and transcriptional features of S100a4+ macrophages were examined across datasets to evaluate their existence under steady‐state or chronic disease conditions.

### 4.15 LC‐MS Untargeted Metabolomics

The samples under different conditions were collected and injected into the LC‐MS/MS system analysis (*n* = 3 biologically independent samples per group). UHPLC‐MS/MS analyses were performed using a Vanquish UHPLC system coupled with an Orbitrap Q Exactive HF mass spectrometer. Samples were injected onto a Hypersil Gold column and separated using a 12‐min linear gradient, with eluents consisting of 0.1% FA in water (A) and methanol (B). The gradient included steps to increase the percentage of B, followed by re‐equilibration. The mass spectrometer operated in positive/negative polarity modes, with a spray voltage of 3.5 kV and capillary temperature of 320°C. Data acquisition was optimized with a resolution of 60 000 at m/z 200.The raw data were processed using Compound Discoverer 3.3 for peak alignment, picking, and quantification. Normalization was performed using total spectral intensity. Peaks were identified by matching with mzCloud, mzVault, and MassList databases. Statistical analyses were conducted using R, Python, and CentOS, with normalization based on relative peak areas in QC samples. Metabolites with coefficients of variation (CV) greater than 30% were excluded. The KEGG, HMDB, and LIPIDMaps databases were used for metabolite annotation. Principal components analysis (PCA) and partial least squares discriminant analysis (PLS‐DA) were performed using metaX software. Univariate analysis (t‐test) identified differential metabolites, with VIP >1, *p*‐value <0.05, and fold change ≥2 or ≤0.5 considered significant. Volcano plots and clustering heat maps were generated to visualize the data. The correlation between differential metabolites was analyzed using Pearson's method. Metabolic pathways were enriched and analyzed for statistical significance using the KEGG database, with pathways having a *p*‐value <0.05 considered significantly enriched.

### 4.16 Proteomics and Data Processing

Liquid chromatography‐tandem mass spectrometry (LC‐MS/MS) analysis was performed using a timsTOF Pro mass spectrometer (Bruker), coupled to an Evosep One liquid chromatography system (Evosep, Denmark) (*n* = 3 biologically independent samples per group). Peptides were first loaded onto a C18 reverse‐phase analytical column equilibrated in buffer A. Separation was achieved using a linear gradient of buffer B at a flow rate of 220 nL/min. The mass spectrometer was operated in positive ion mode with an electrospray voltage of 1.6 kV. Precursor ions and their corresponding fragments were analyzed using a time‐of‐flight (TOF) detector across a mass range of m/z 100–1700. Data acquisition was performed in parallel accumulation serial fragmentation (PASEF) mode, enabling rapid collection of MS and MS/MS scans. For each PASEF cycle, 1 MS scan was followed by 8 MS/MS scans. The ion mobility coefficient (1/K0) was set between 0.75 and 1.35 Vs/cm^2^ to resolve ions in the gas phase prior to fragmentation. Active exclusion of previously fragmented precursors was applied to improve data acquisition efficiency, with a release time of 24 s. Raw data were processed and analyzed using MaxQuant software (v1.6.0.1), with default settings for database searching against the mouse reference proteome from UniProt. MaxQuant settings included a fixed modification of carbamidomethylation on cysteine residues and variable modifications for oxidation (M) and acetylation (protein N‐terminal). The false discovery rate (FDR) was set to 1% at the peptide‐spectrum match (PSM) and protein levels. Protein quantification was achieved through the MaxLFQ algorithm, with an LFQ minimum ratio count of 2 to ensure reliable quantitation across replicates. After identification and quantification, normalization was performed using a log2 transformation of the LFQ intensity values to correct for technical variation.

### 4.17 Multicolor Immunofluorescence and Co‐localization Analysis

To generate FITC‐labeled PW (PW‐FITC), PW nanoparticles were first synthesized as described above and subsequently conjugated with fluorescein isothiocyanate (FITC) by incubation at 4°C under gentle stirring for 6 h. Excess free FITC was removed by repeated washing and centrifugation. Primary rat macrophages were seeded and incubated with PW‐FITC for 24 h. After treatment, cells were fixed with 4% paraformaldehyde for 20 min at room temperature, permeabilized with 0.3% Triton X‐100 in PBS for 30 min, and blocked with 5% bovine serum albumin (BSA) for 1 h. Cells were then incubated overnight at 4°C with a primary antibody against HK2 (Abcam, #EPR20839). Following washing, cells were incubated with an Alexa Fluor 555‐conjugated secondary antibody (Abcam, #ab150078) for 2 h at room temperature. Nuclei were counterstained with DAPI. Fluorescence images were acquired using a Zeiss confocal laser scanning microscope under identical imaging settings across groups. Co‐localization of PW‐FITC (green) and HK2 (red) signals was visualized in merged images, and quantitative co‐localization analysis was performed by Image J.

### 4.18 Co‐Immunoprecipitation of PW‐FITC and HK2 in LPS Stimulated Primary Macrophages

Primary rat macrophages were stimulated with LPS to induce an inflammatory phenotype and subsequently incubated with FITC‐labeled PW (PW‐FITC) for 12 h at 37°C. Following incubation, cells were thoroughly washed with cold PBS to remove unbound PW‐FITC and lysed in IP lysis buffer supplemented with protease inhibitors. The cell lysates were incubated overnight at 4°C with an anti‐FITC antibody (Thermo Fisher Scientific, #71‐1900) to capture PW‐FITC‐associated complexes. Antibody–antigen complexes were then enriched using magnetic beads (Selleck, B23201) according to the manufacturer's instructions. After extensive washing to minimize nonspecific binding, the bound proteins were eluted and subjected to SDS‐PAGE and immunoblotting. HK2 was detected using an anti‐HK2 antibody (Abcam, clone EPR20839).

### 4.19 Enzyme‐Linked Immunoassay

To detect the metabolites associated with the norepinephrine synthesis pathway, tumor cells with or without Bap1 deficiency were plated at a concentration of 1.5 × 10^5^ cells/mL for 48 h. Subsequently, the cell culture supernatants were collected. For serum samples, the blood samples were collected and centrifuged at 2,000 × g for 30 min at 4°C. After centrifugation, carefully collect the clear serum for further analysis. The concentrations of different metabolites in the supernatants or serums were measured using corresponding ELISA kits. These kits included those for CCL2 Elisa kit (multi sciences, #EK387), atty acid uptake assay kit (dojindo, #UP07), glucose uptake assay kit‐green (dojindo, #UP02), α‐ketoglutaric acid (mlbio, #YJ028396‐96T) and fatty acid β‐oxidase (mlbio, #ml059584), in accordance with the manufacturer's instructions. Three replicates were set for each cell supernatant sample.

### 4.20 Flow Cytometry

Bone marrow was harvested from the femur or tibia of experimental rats by cutting both ends of the bones and flushing the marrow cavity with PBS using a syringe. The bone marrow suspension was passed through a 70 µm filter to remove debris and large tissue fragments. For surface staining, cells were incubated with the appropriate antibodies at room temperature for 30 min, followed by a wash with DPBS (500 g for 10 min). Fatty acid or glucose uptake assay was determined by fatty acid uptake assay kit (dojindo, #UP07) and glucose uptake assay kit ‐green (dojindo, #UP02), in accordance with the manufacturer's instructions.

To analyze the cell cycle distribution, Schwann cell under different conditions were harvested as described above and fixed in 70% ethanol at 4°C overnight. After fixation, cells were washed with PBS and incubated with RNase A (50 µg/mL) at 37°C for 30 min to degrade RNA. Propidium iodide (PI) was then added (50 µg/mL) to stain DNA. The stained cells were analyzed using a flow cytometer, and the DNA content was measured to determine the cell cycle phases (G0/G1, S, and G2/M). Data were analyzed using appropriate software to quantify the percentage of cells in each phase of the cycle.

### 4.21 S100a4^+^ Macrophage Sorting

Bone marrow mononuclear cells were isolated from mice and processed into a single‐cell suspension. Cells were cultured in complete medium supplemented with 20 ng/mL macrophage colony‐stimulating factor (M‐CSF) at 37°C with 5% CO_2_ for 5 days to promote differentiation into bone marrow‐derived macrophages (BMDMs). After differentiation, BMDMs were collected and incubated with a mixture of primary antibodies, anti‐CD169 (anti‐mouse) and anti‐ATP1A3 (anti‐rabbit), which were highly expressed in S100a4^+^ macrophages, for 30 min at 4°C in the dark. Cells were subsequently stained with species‐specific fluorescent secondary antibodies, Goat Anti‐Mouse‐AF594 and Goat Anti‐Rabbit‐AF488, for an additional 30 min. Following incubation, cells were washed with PBS, resuspended in FACS buffer, and filtered through a 40 µm cell strainer to remove aggregates. Flow cytometric analysis and sorting were then performed to isolate the CD169^+^ATP1A3^+^ double‐positive macrophage population.

### 4.22 Schwann Cell Sorting

Single cells suspension was harvested as described above, washed with PBS and resuspended in FACS buffer (PBS supplemented with 1% fetal bovine serum). To identify Schwann cells, the suspension was incubated with rabbit anti‐p75NTR primary antibody for 30 min at 4°C in the dark. After washing, cells were incubated with an Alexa Fluor 488‐conjugated goat anti‐rabbit secondary antibody for an additional 30 min. Following incubation, cells were washed with PBS, resuspended in FACS buffer, and filtered through a 40 µm cell strainer to remove aggregates. Flow cytometric analysis and sorting were then performed to isolate the p75NTR^+^CD45‐Schwann cell population.

### 4.23 Extracellular Flux Assay

Real‐time bioenergetic profiles of BMDMs were determined by measuring the oxygen consumption rate (OCR) and extracellular acidification rate (ECAR) using the XFe96 Extracellular Flux Analyzer with Wave v.2.4.2 (Seahorse Bioscience, Agilent Technologies). BMDMs (80 000 cells) were pretreated with 10 µM RXD or vehicle for 1 h, followed by treatment with PBS, PW (5 ppm), and/or lipopolysaccharide (LPS) (100 ng/mL) for 24 h. Assays were performed according to the manufacturer's protocol. In brief, the Cell Mito Stress Test was performed by sequential titration with 1 µm oligomycin, 1.5 µm carbonylcyanide 4‐(trifluoromethoxy) phenylhydrazone (FCCP), and 0.5 µM rotenone/antimycin A. A Glyco Stress Test was conducted by sequential titration with 10 mm glucose, 1 µm oligomycin, and 50 mm 2‐deoxyglucose. Mito Fuel Flex Tests were performed by adding 3 µm BPTES or 2 µm UK5099 (provided in the test kit, Agilent) after measuring baseline respiration.The basal and maximal respiration, as well as glycolysis and glycolytic capacity, were calculated following the manufacturer's recommendations.

### 4.24 Western Blot

Protein samples (25 µg) were resolved by SDS‐polyacrylamide gel electrophoresis (SDS‐PAGE) using a Bio‐Rad system (Bio‐Rad, Hercules, CA). The electrophoresed proteins were then transferred onto a polyvinylidene difluoride (PVDF) membrane (Bio‐Rad). After three washes, the membranes were blocked with 5% milk in PBS for 1 h at room temperature and then incubated overnight at 4°C with primary antibodies diluted 1:1000. The antibodies targeted H3K4me3 (CST, #9751), H3K9me3 (Abcam, #ab176916), H3K27me3 (CST, #9733), Histone H3 (Invitrogen, #PA516183), and β‐actin (CST, #4967). Following three additional washes, the membranes were incubated with enzyme‐conjugated, species‐specific secondary antibodies for 1 h at room temperature. Immunoreactive bands were visualized using SuperSignal West Dura Extended Duration Substrate (Pierce Biotechnology).

### 4.25 Chip‐qPCR

BMDMs under different conditions were crosslinked with 1% formaldehyde for 8 min at room temperature and quenched with glycine. Following cell lysis, the chromatin was fragmented into 100–500 bp using a Bioruptor Pico (Diagenode, Belgium). Protein‐DNA complexes were immunoprecipitated (IP) using H3K9me3 (Abcam, #ab176916) or anti‐IgG antibodies (Cell Signaling Technology, MA, USA) and Dynal magnetic beads (Cell Signaling Technology, MA, USA) on a rotator at 4°C overnight.

After washing and reversal of crosslinks, ChIP‐DNA was purified following a standard protocol using the QIAquick Gel Extraction Kit (QIAGEN, Hilden, Germany). The IP and input DNA were then amplified by quantitative PCR (qPCR) with primers targeting sequences near the putative binding sites in the S100a4 or Fn1 promoter regions.

### 4.26 CUT&Tag

BMDMs stimulated with LPS and treated with or without PW were collected and permeabilized to allow access of the H3K9me3 antibody (Abcam, #ab176916) for sequencing (Shanghai Neobio Biotech). Raw sequencing reads were processed using Trimmomatic (v0.35) for data filtering. Alignment was performed with BWA software (v0.7.17). Fragment sizes for paired‐end reads were calculated from the BAM files generated by the aligned sequencing data. Summary statistics on fragment lengths were obtained by sampling several regions, depending on genome size and processor availability. Peak calling was conducted using MACS2 (v2.2.7.1), and peak annotation analysis was primarily performed with Bedtools (v2.30.0).

### 4.27 Statistical Analysis

Statistical analyses were performed using R version 4.0.3 or GraphPad Prism 9.5. Comparisons between two groups were performed using a two‐tailed unpaired *t*‐test. A one‐way ANOVA with Tukey's post‐hoc test was employed for the purpose of comparing the effects of multiple levels of a single factor. Enrichment scores derived from ssGSEA were compared between groups using non‐parametric tests (Wilcoxon rank‐sum test) to account for potential non‐normal distribution of scores. Differential expression analyses between BMDM_LPS and BMDM_LPS_PW samples were performed using the DESeq2 package (v1.30.1), with p‐values adjusted for multiple testing using the Benjamini–Hochberg method. A minimum of five rats per group was included to ensure sufficient statistical power. For in vitro experiments, at least three biological replicates were used unless otherwise specified. For differential expression analysis, *p* values were adjusted for multiple comparisons using the Benjamini–Hochberg method. Statistical significance was defined as a *p* value or adjusted *p* value <0.05.

## Funding

National Natural Science Foundation of China (82030050, T2394534, to Y.Z.; 82172074, 82572389 to X.C.).

## Conflicts of Interest

The authors declare no conflicts of interest.

## Supporting information




**Supporting File**: advs74504‐sup‐0001‐SuppMat.docx.

## Data Availability

The raw sequencing data presented in this paper have been deposited in the Genome Sequence Archive at the National Genomics Data Center (Beijing, China) under BioProject ID PRJCA036111. These files are accessible under controlled access and can be obtained by submitting a request to the Data Access Committee.
