# Supplementary material for: Nanoparticle‐Mediated Immunometabolic‐Epigenetic Remodeling Enhances Schwann Cell‐Macrophage Interaction for Sciatic Nerve Regeneration
Source: Adv Sci (Weinh). 2026 Feb 20;13(25):e22093. doi: 10.1002/advs.202522093 (PMC13137827; doi:10.1002/advs.202522093)
Supplement: Supplementary file 1 — Supporting File: advs74504‐sup‐0001‐SuppMat.docx. [file ADVS-13-e22093-s001.docx]

Supporting Information

**Nanoparticle-Mediated Immunometabolic-Epigenetic Remodeling Enhances Schwann Cell-Macrophage Interaction for Sciatic Nerve Regeneration**

*Wenying Xu^1, 2^, Rongrong Wu^1, 2^, Zhicheng Liu^3^, Hongyu Yan^1, 2^, Huan Zhang^1, 2^, Yuanyi Zheng^1, 2, *^, Xiaojun Cai^1, 2, *^*

^1^ Shanghai Key Laboratory of Neuro-Ultrasound for Diagnosis and Treatment, Shanghai Sixth People’s Hospital Affiliated to Shanghai Jiao Tong University School of Medicine, Shanghai, China

^2^ Department of Ultrasound in Medicine, Shanghai Sixth People's Hospital Affiliated to Shanghai Jiao Tong University School of Medicine, Shanghai, China

^3^ Hepatic Surgery Center, Tongji Hospital, Tongji Medical College, Huazhong University of Science and Technology, Wuhan, China

*Corresponding authors. E-mail: [c1x2j34@163.com](mailto:c1x2j34@163.com) or [caixiaojun00@sjtu.edu.cn](mailto:caixiaojun00@sjtu.edu.cn); [zhengyuanyi@sjtu.edu.cn](mailto:zhengyuanyi@sjtu.edu.cn).


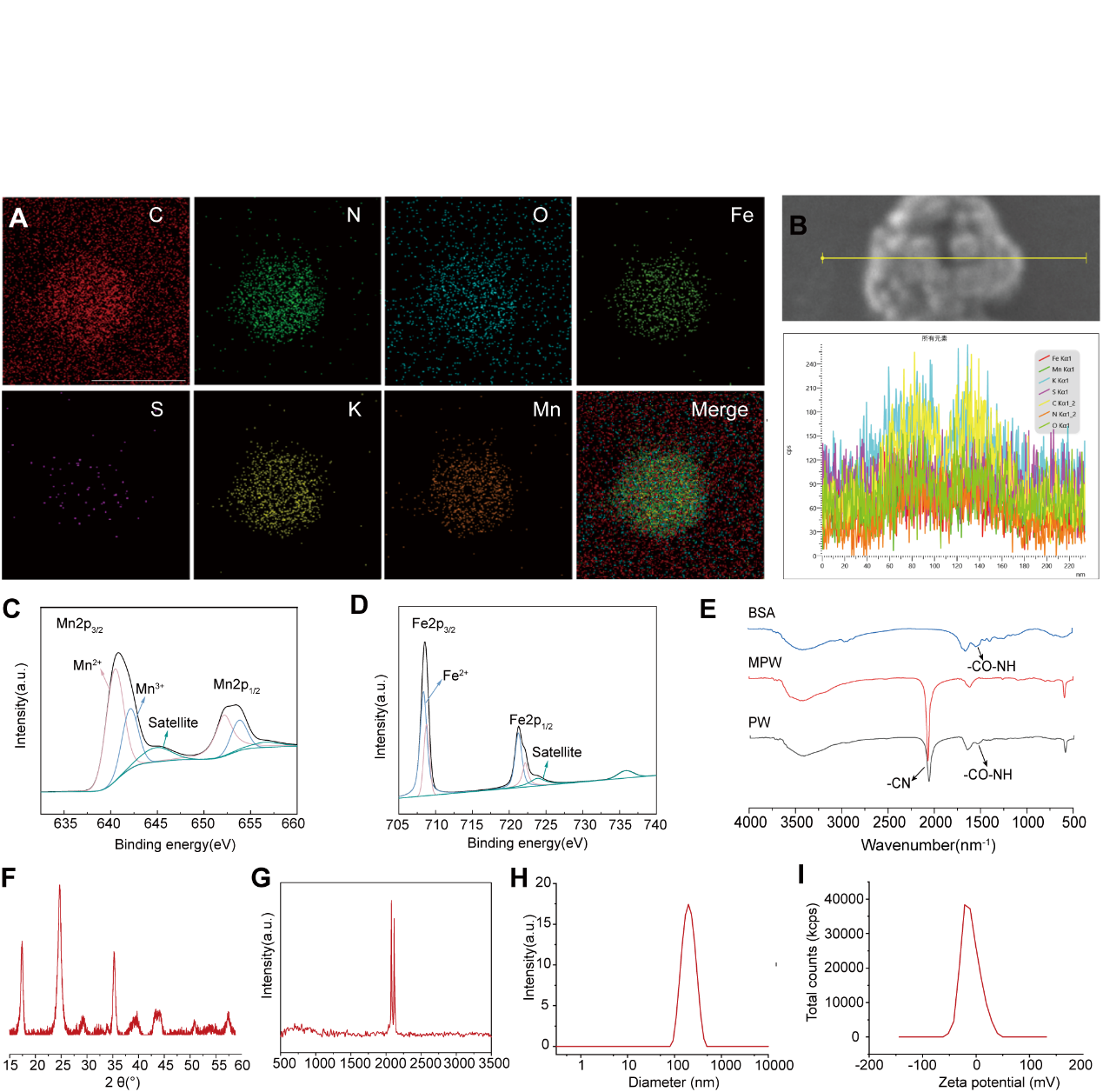


**Figure S1. Comprehensive physicochemical characterization of Prussian White nanoparticles (PW). (A)** Elemental mapping confirming the presence of C, K, Mn, Fe, N, and S in PW. **(B)** Spatial distribution of C, N, O, Fe, S, K, and Mn elements, with merged image demonstrating compositional homogeneity. **(C-D)** X-ray photoelectron spectroscopy (XPS) spectra of Fe 2p and Mn 2p core levels, confirming the coexistence of Fe^2+^/Fe^3+^ and Mn^2+^ oxidation states. **(E)** Fourier-transform infrared (FTIR) spectra of BSA, MPW (MnCl_2_-K_4_[Fe(CN)_6_]), and PW, indicating characteristic –C≡N– and amide (CO–NH) vibrations. **(F, G)** X-ray diffraction (XRD) patterns **(F)** and Raman spectra **(G)** confirming the crystalline structure and cyanide vibrational modes of PW. **(H, I)** Dynamic light scattering (DLS) size distribution **(H)** and zeta potential analysis **(I)**, indicating uniform nanoscale dimensions and colloidal stability.


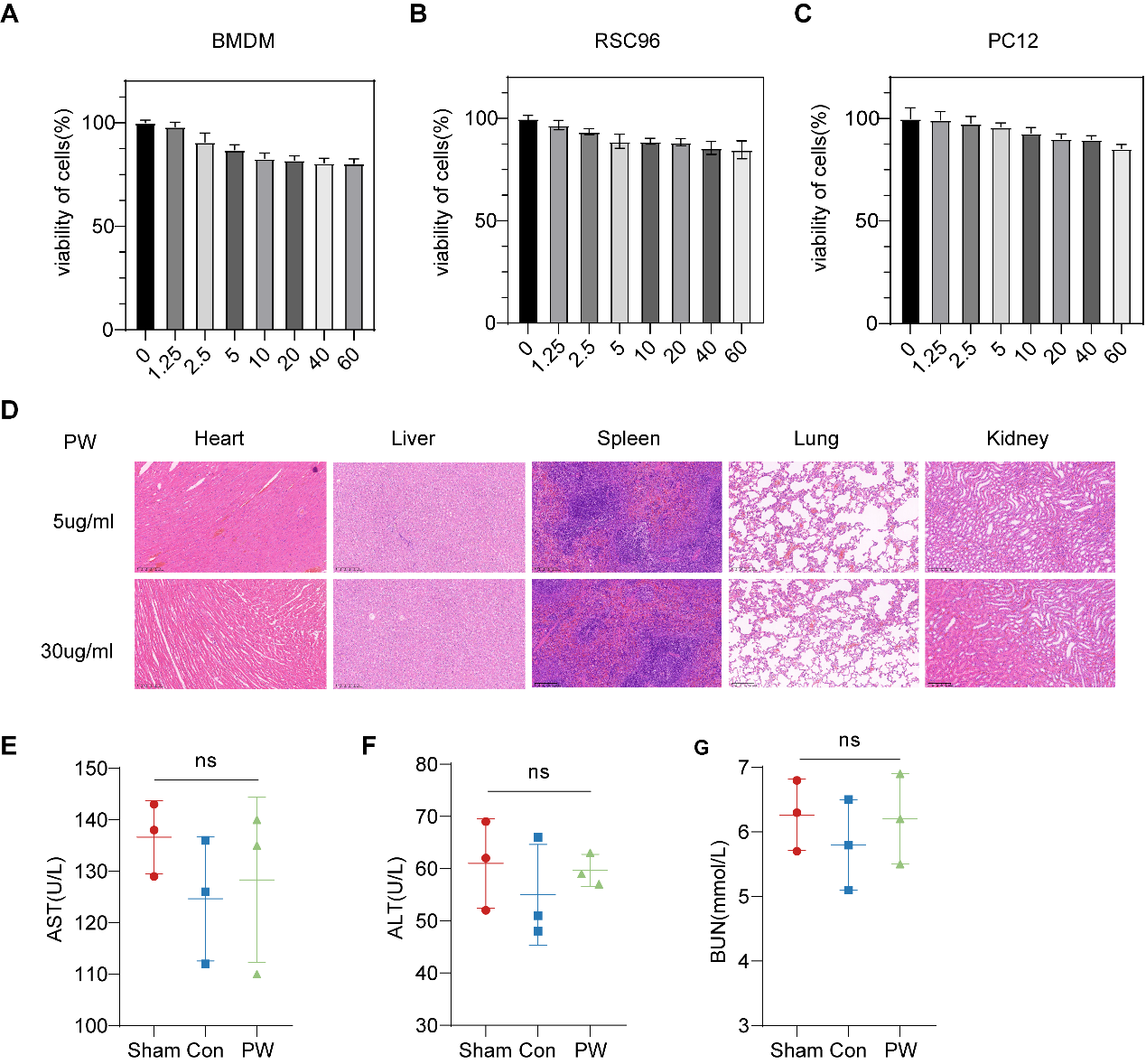


**Figure S2. In vitro and in vivo biosafety assessment of PW. (A-C)** Cytotoxicity evaluation of PW at varying concentrations (1.25-60 μg/mL) in bone marrow-derived macrophages (BMDMs) **(A)**, RSC96 Schwann cells **(B)**, and PC12 neuronal cells **(C)**. **(D)** Hematoxylin and eosin (H&E) staining of major organs 4 weeks post-PW treatment at 30 μg/mL and 5 μg/mL (n = 3/group). Scale bars: 200 μm. **(E-G)** Serum levels of AST **(E)**, ALT **(F)**, and BUN **(G)** in rats 4 weeks post-treatment (n = 3/group). Mean values are shown and error bars represent ± s.d., as analyzed by one-way ANOVA with Tukey’s post hoc tests in (**E-F**). ns, not significant.


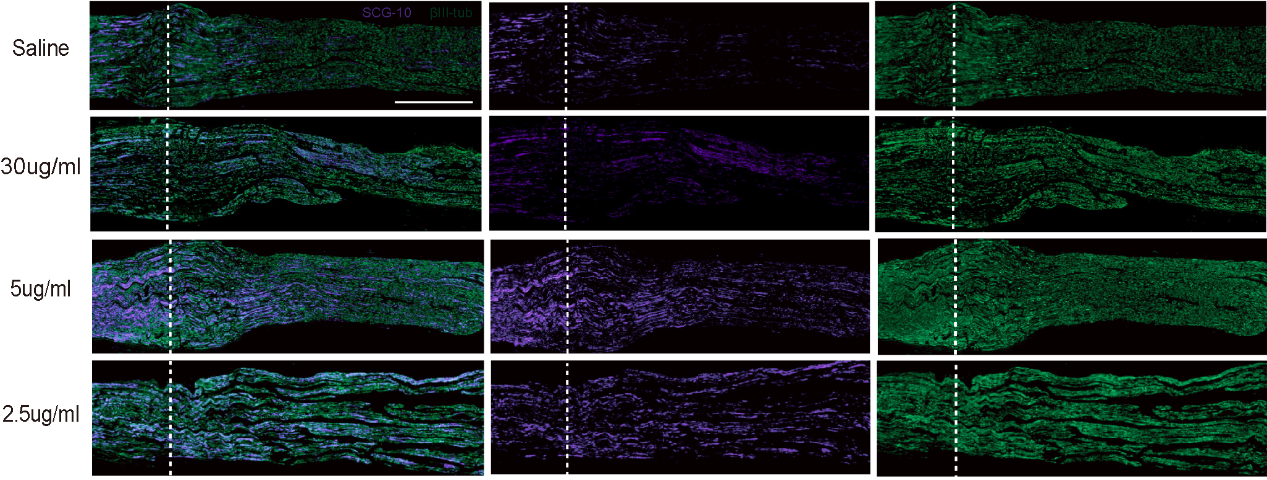


**Figure S3. Optimization of PW dosage for promoting axonal regeneration after SNI.** Longitudinal sections of sciatic nerves 14 days post-SNI, immunostained for SCG-10 (purple) and βIII-tubulin (green), with DAPI (blue) for nuclei. Treatments include saline (Con) and PW at 30, 5, and 2.5 μg/mL. Scale bar: 1 mm.


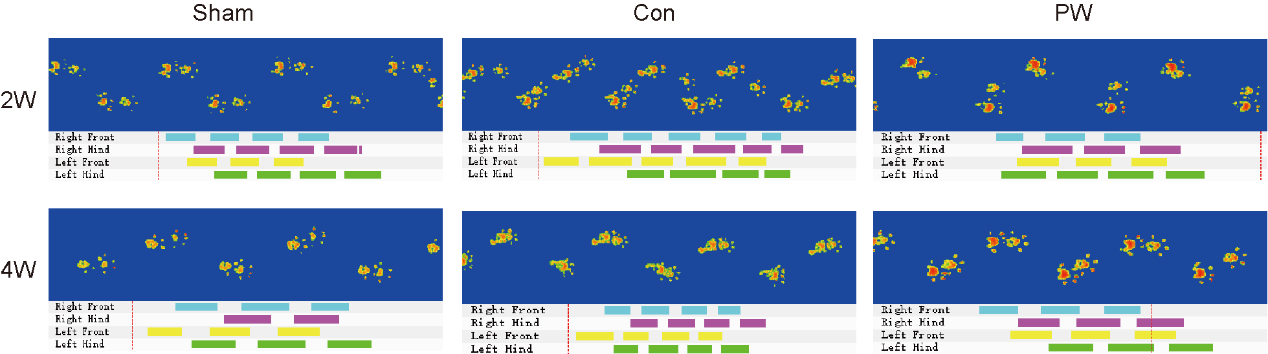


**Figure S4. Gait analysis of SNI rats using the CatWalk system.** Paw print patterns and gait parameters assessed at 2 and 4 weeks post-injury in Sham, saline (Con), and PW-treated groups.


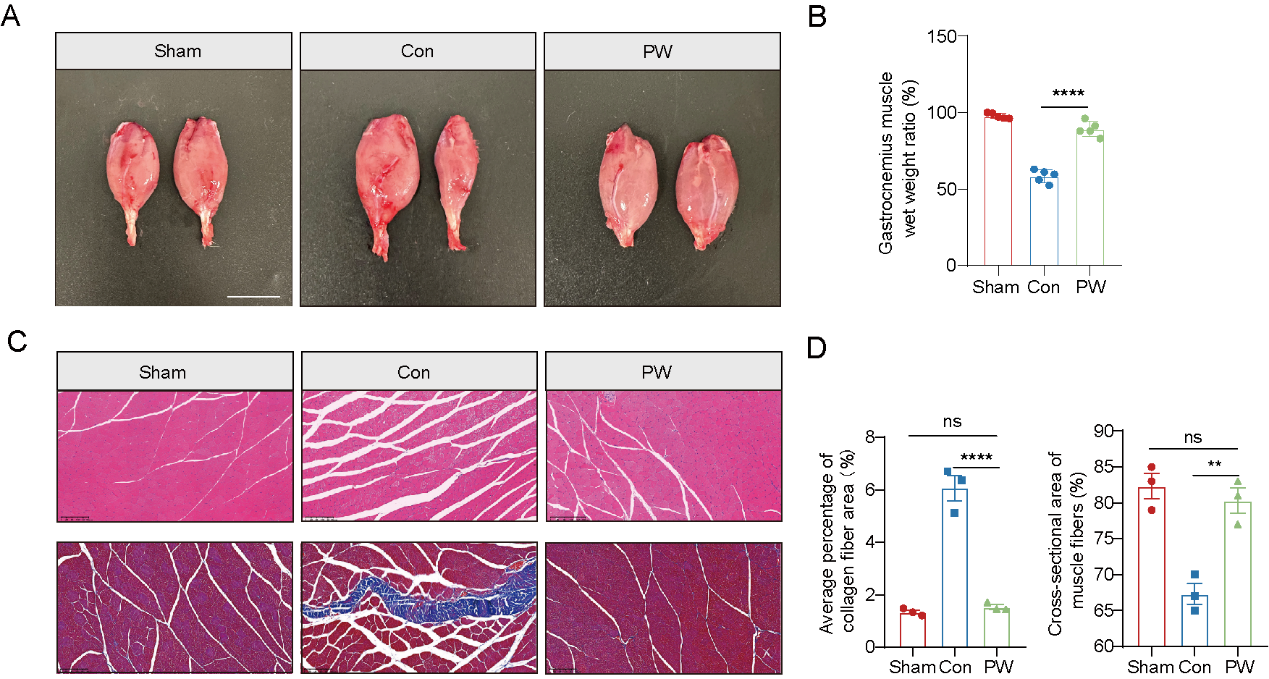


**Figure S5. PW mitigates denervation-induced muscle atrophy and fibrosis. (A)** Gross morphology of gastrocnemius muscles. Scale bars: 1 mm. **(B)** Muscle mass normalized to body weight (n = 5/group). **(C)** H&E (upper) and Masson’s trichrome (lower) staining of muscle sections. Scale bars: 200 μm (upper), 10 μm (lower) **(D)** Quantification of atrophied fiber percentage and cross-sectional area (n = 3/group). Mean values are shown and error bars represent ± s.d., as analyzed by one-way ANOVA with Tukey’s post hoc tests in (B) and (D).ns, not significant; **P < 0.01, ****P < 0.0001.


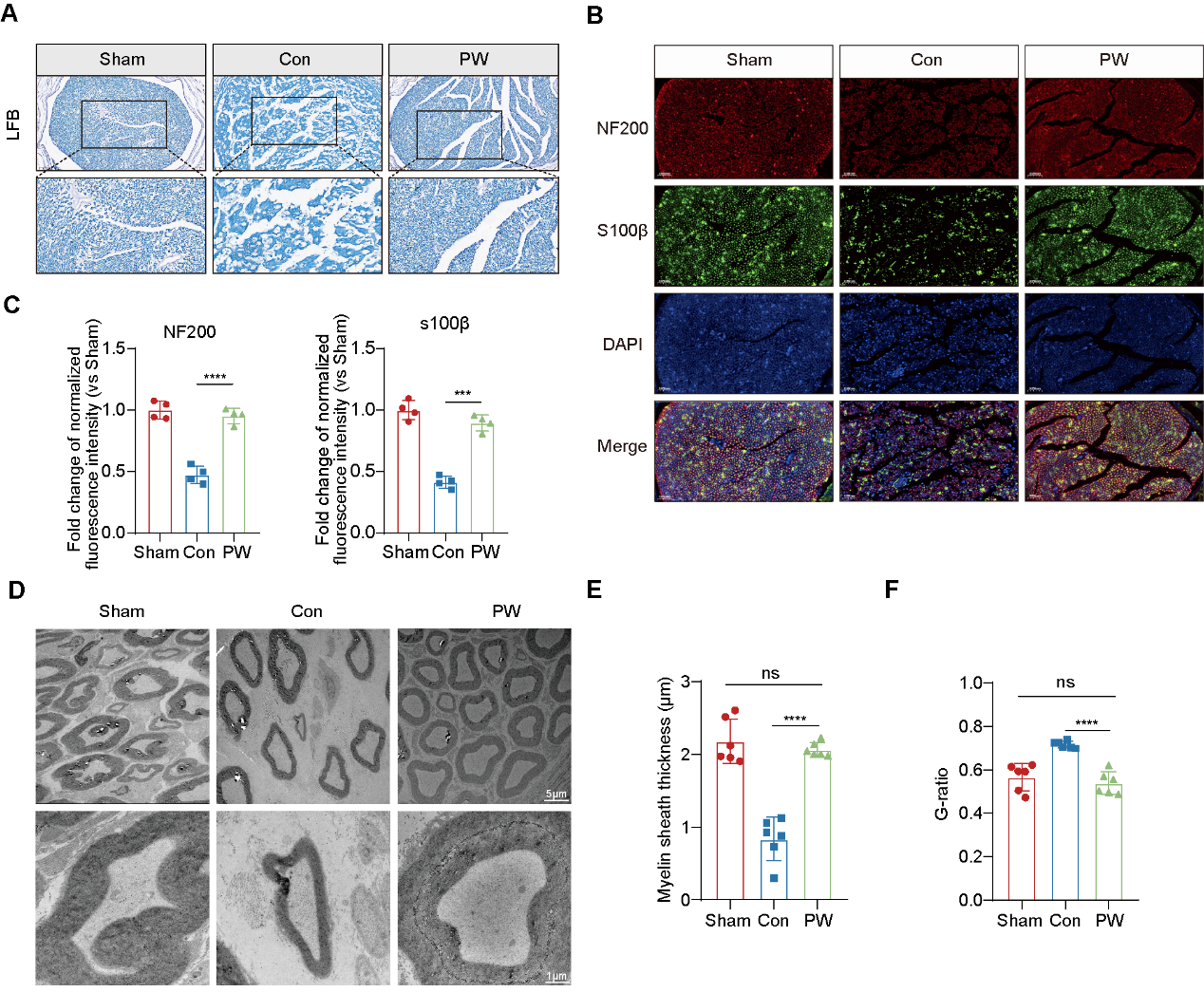


**Figure S6. PW enhances myelination and ultrastructural recovery. (A)** Luxol Fast Blue (LFB) staining of sciatic nerve sections. Scale bars: 100 μm (upper), 50 μm (lower) **(B, C)** Multiplex immunohistochemistry (mIHC) for NF200 (an indicator for axons) and S100β (an indicator for Schwann cells) **(B)** and quantitative analysis **(C)** (n = 4/group). Scale bars: 50 μm. **(D)** TEM images of nerve cross-sections. **(E, F)** Quantification of myelin sheath thickness **(E)** and g-ratio **(F)**. Two regions per sample (n = 3/group). Scale bars: 5 μm (upper), 1 μm (lower). Mean values are shown and error bars represent ± s.d., as analyzed by one-way ANOVA with Tukey’s post hoc tests in (C), (E) and (F). ns, not significant; ***P < 0.001, ****P < 0.0001.


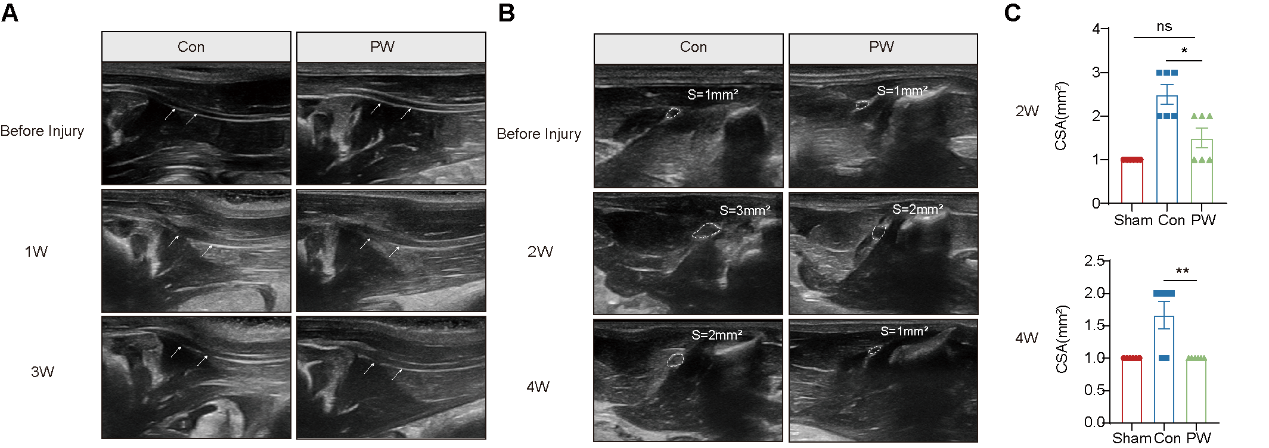


**Figure S7. Ultrasonographic monitoring of sciatic nerve recovery. (A)** Representative longitudinal nerve ultrasound images at baseline, 1 week (1W), and 3 weeks (3W) post-injury.. **(B)** Cross-sectional area (CSA) measurements at baseline, 2 weeks (2W), and 4 weeks (4W). **(C)** Quantification of CSA at 2W and 4W (n = 6/group). Mean values are shown and error bars represent ± s.d., as analyzed by one-way ANOVA with Tukey’s post hoc tests in (C). ns, not significant; *P < 0.05, **P < 0.01.


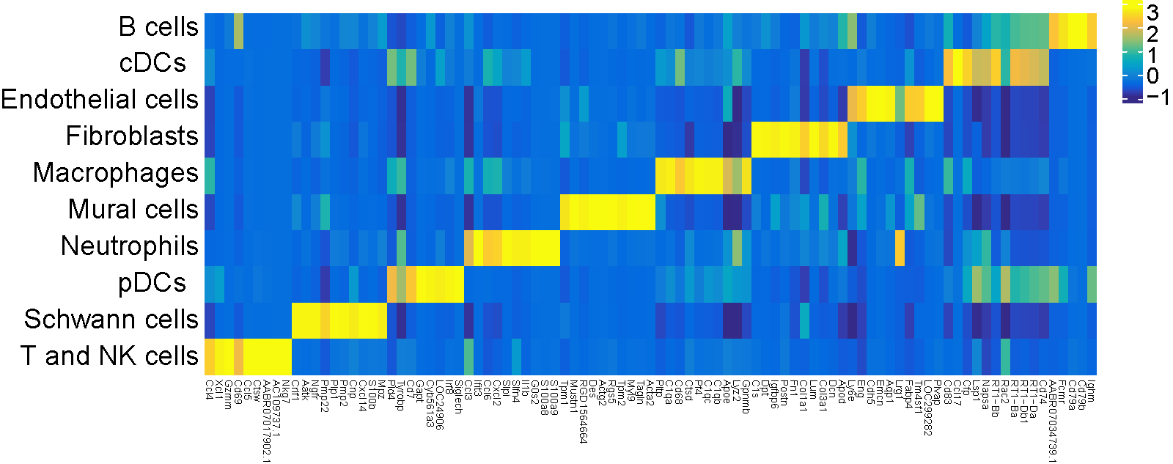


**Figure S8. Single-cell RNA sequencing identifies major cell types in sciatic nerve tissue.** Heatmap of marker gene expression for B cells, dendritic cells, endothelial cells, fibroblasts, macrophages, mast cells, neutrophils, Schwann cells, and T/NK cells.


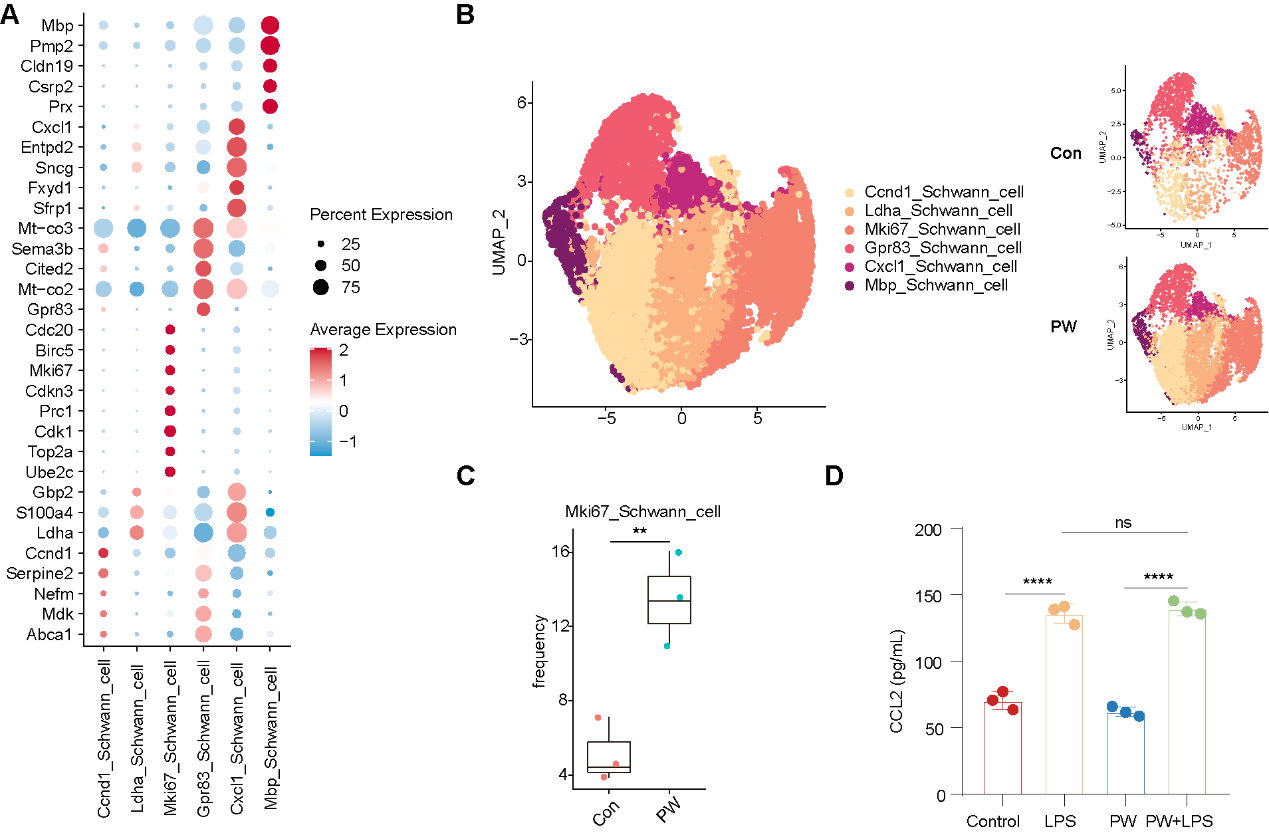


**Figure S9. PW modulates Schwann cell proliferation and macrophage-Schwann cell crosstalk. (A)** Dot plot of marker gene expression in macrophage subpopulations. **(B)** UMAP and density plots of Schwann cell distribution. **(C)** Frequency of Mki67^+^ Schwann cells (n = 3/group). **(D)** CCL2 concentration in Schwann cells across groups (n = 3/group). Mean values are shown and error bars represent ± s.d., as analyzed by t unpaired Student’s t test in (**C**), or one-way ANOVA with Tukey’s post hoc tests in (**D**). ns, not significant; **P < 0.01, ****P < 0.0001.


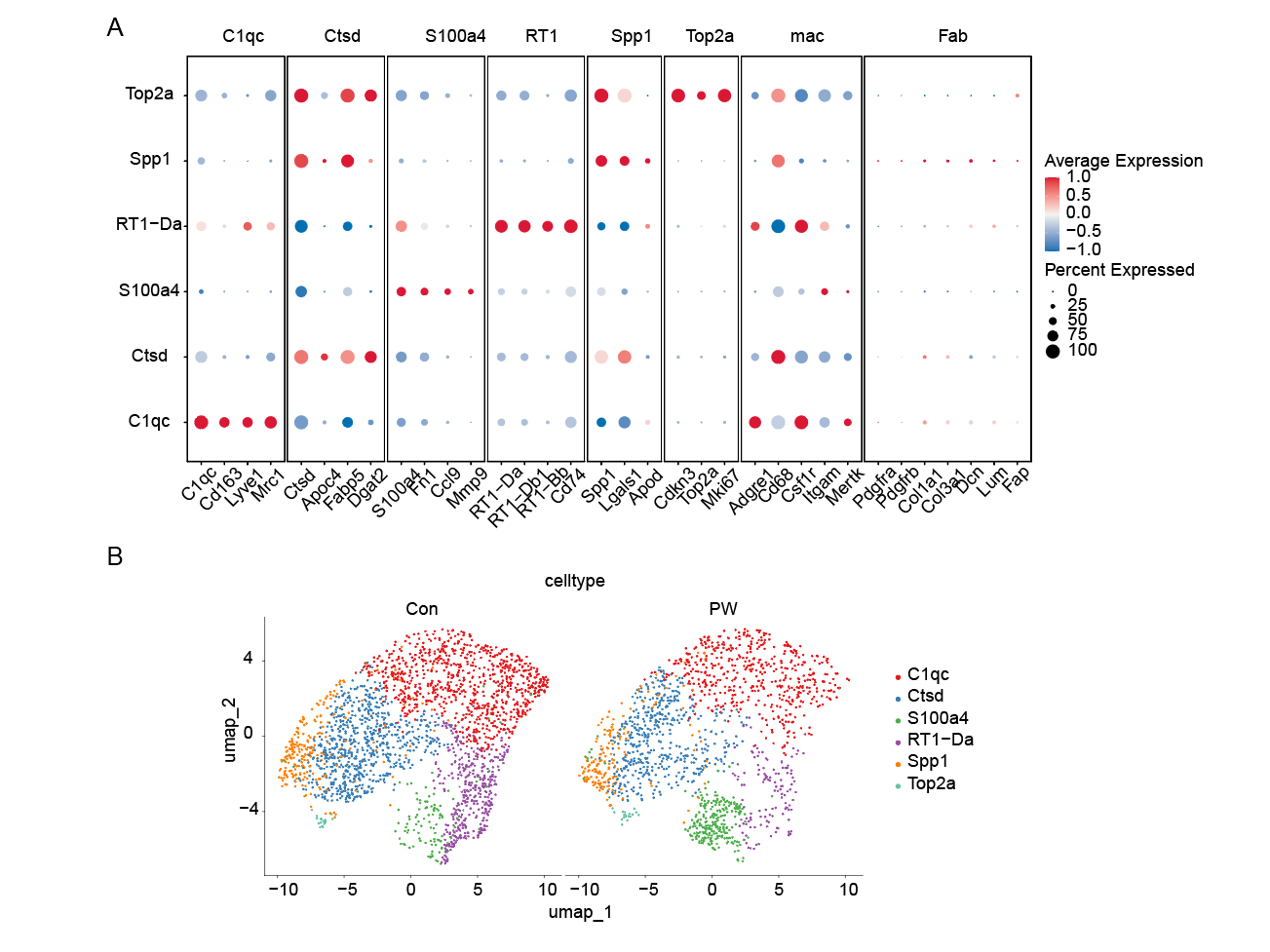


**Figure S10. Macrophage subpopulation characterization. (A)** Marker gene expression profiles defining the S100a4⁺ macrophage subpopulation distinct from other macrophage subsets or fibroblast. **(B)** Scatter plot of marker gene expression in macrophage subpopulations.


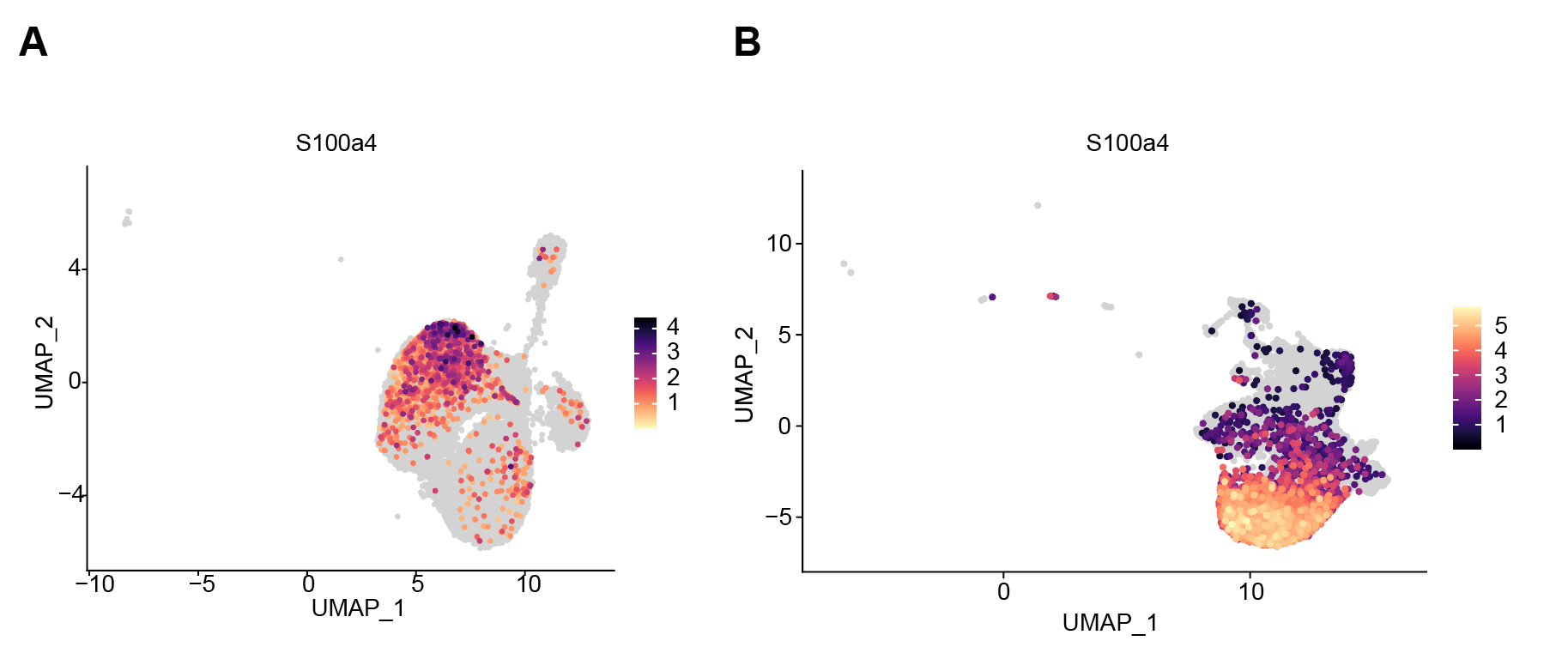


**Figure S11.** The UMAP visualization of single-cell RNA sequencing data from a publicly available dermatomyositis (**A**) or HCC (**B**) dataset demonstrates the presence of S100a4⁺ macrophage subpopulation.


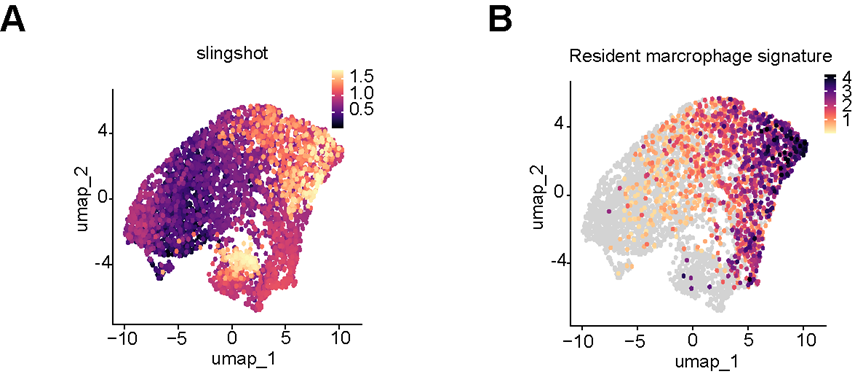


**Figure S12. Pseudotime trajectory analysis of macrophage differentiation. (A)** Slingshot trajectory analysis revealing distinct macrophage lineage differentiation. **(B)** Resident macrophage signature scores indicating bone marrow-derived vs. resident origins.


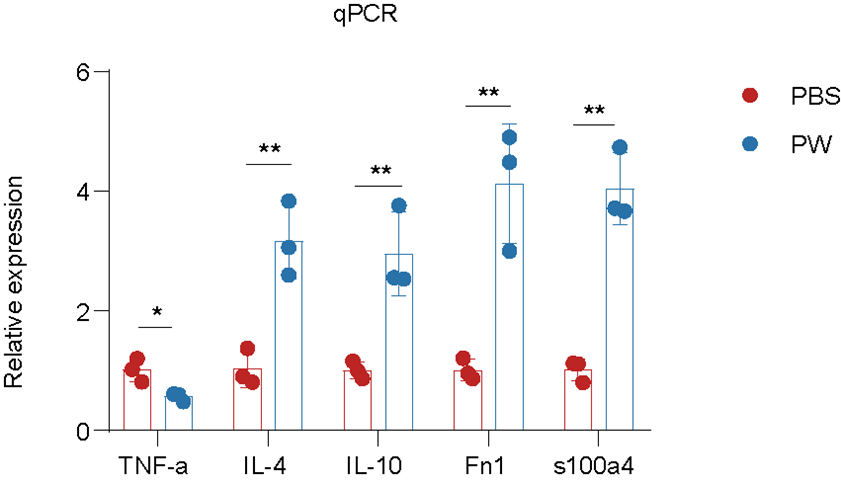


**Figure S13. PW promotes anti-inflammatory and pro-regenerative gene expression in macrophages.** qRT-PCR analysis of TNF-α, IL-4, IL-10, Fn1, and S100a4 in control and PW-treated BMDMs (n = 3). Mean values are shown and error bars represent ± s.d., as analyzed by t unpaired Student’s t test. *P < 0.05, **P < 0.01.


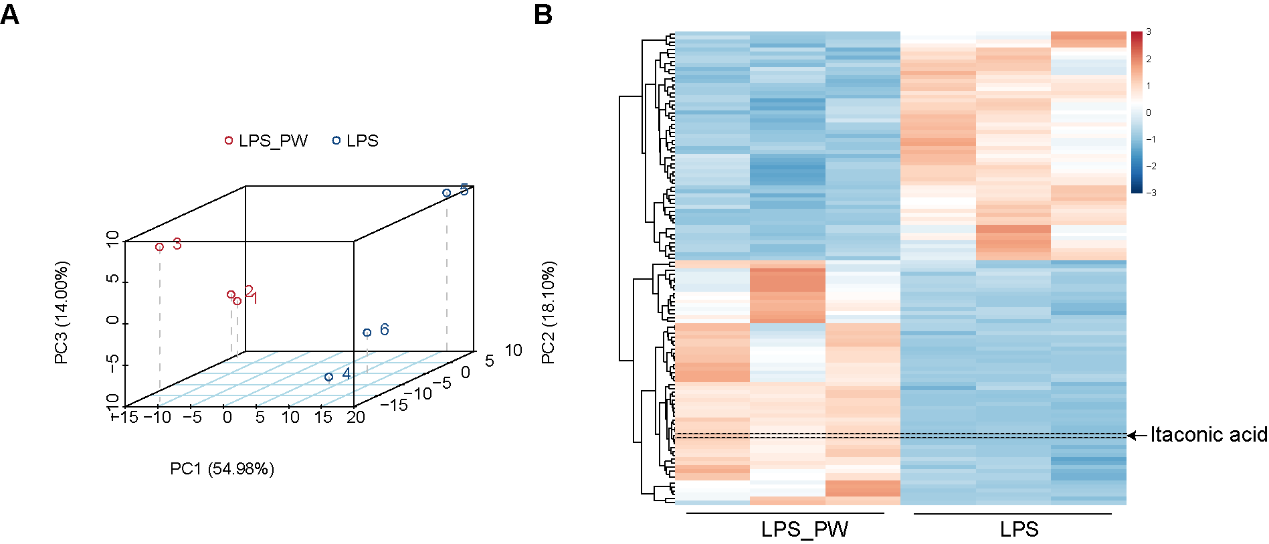


**Figure S14. Metabolomic profiling of BMDMs reveals PW-induced metabolic shifts. (A)** Principal component analysis (PCA) of metabolomic profiles from LPS and LPS_PW groups. **(B)** Heatmap of differentially expressed metabolites, highlighting itaconate upregulation in LPS_PW-treated BMDMs.


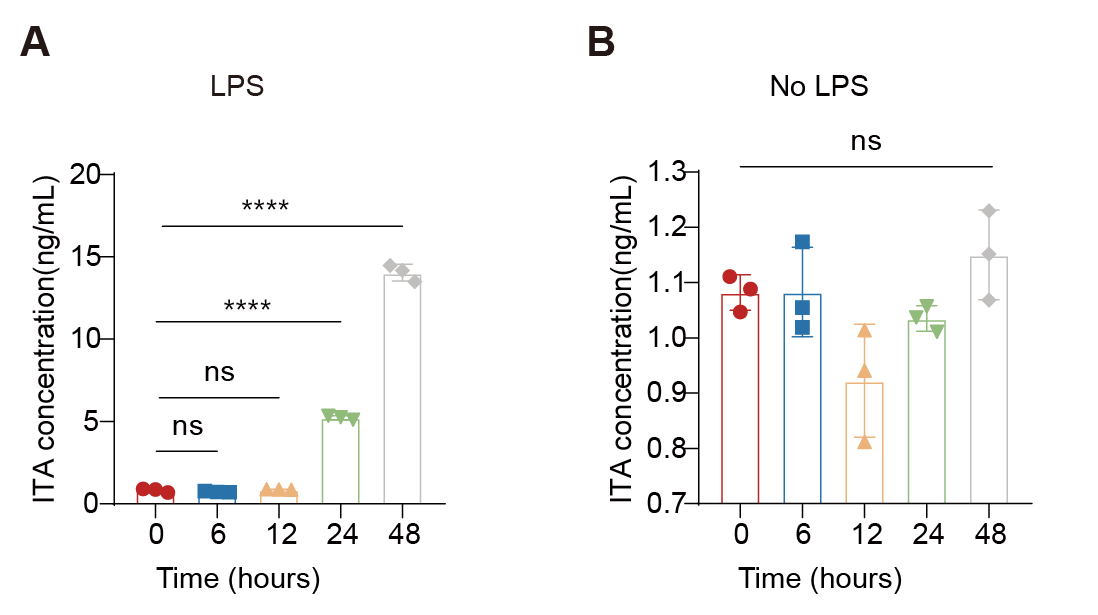


**Figure S15.** ELISA measurement of itaconate (ITA) levels in the supernatants of LPS-stimulated (**A**) or no LPS-stimulated (**B**) macrophages at different time points after PW treatment (n = 3/group). Mean values are shown and error bars represent ± s.d., as analyzed by one-way ANOVA with Tukey’s post hoc tests in (**A**) and (**B**). ns, not significant; ****P < 0.0001.


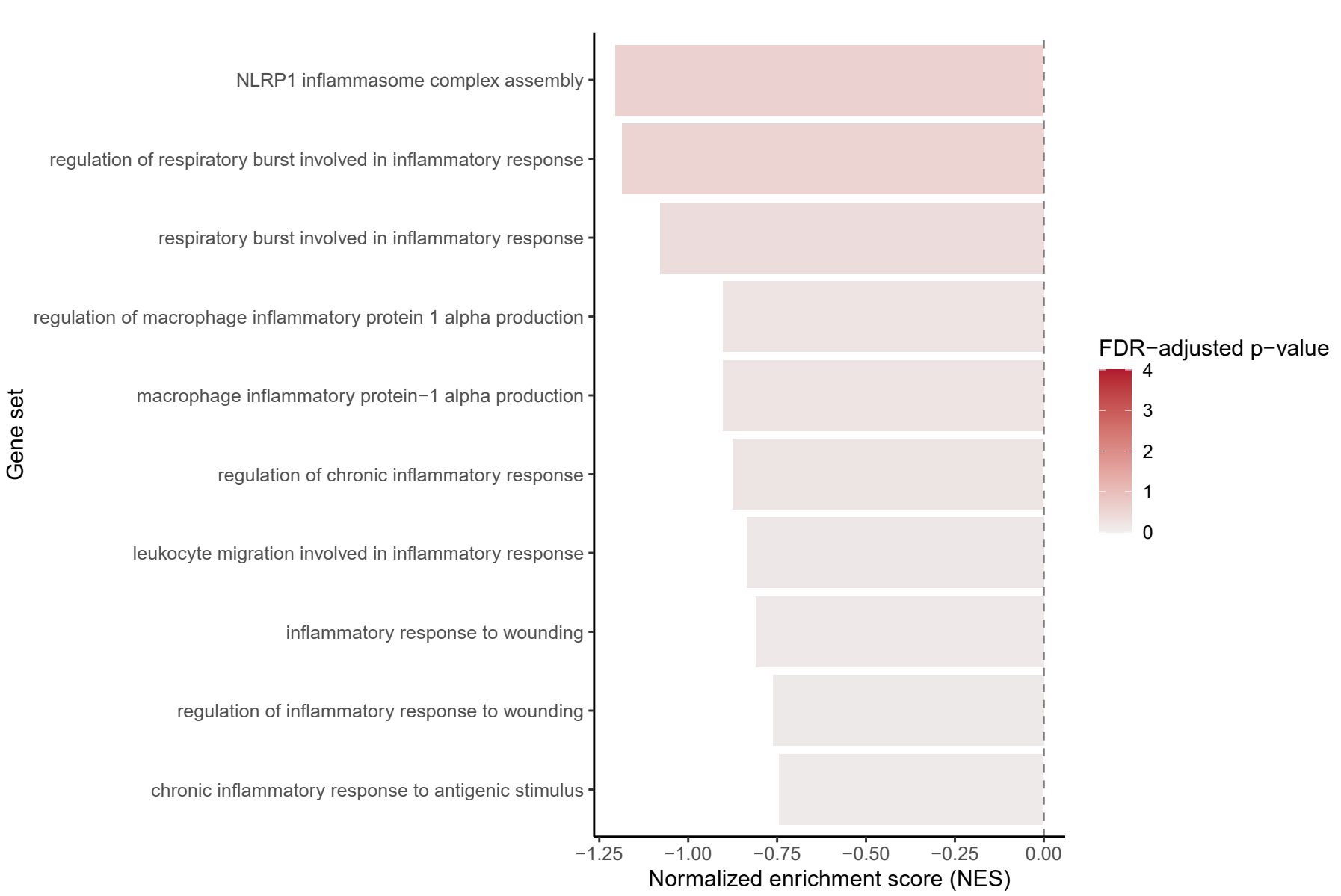


**Figure S16.** Gene set enrichment analysis (GSEA) of bulk RNA-seq data showing Itaconate attenuates inflammation-associated gene programs in Schwann cells.


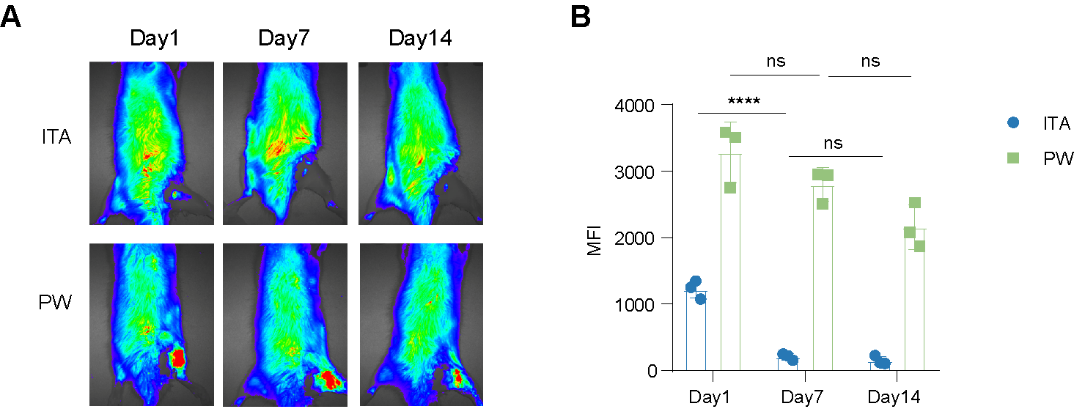


**Figure S17. In vivo biodistribution and retention of PW and free itaconate (ITA). (A)** Fluorescence imaging of Cy5.5-labeled ITA and PW over 14 days. **(B)** Quantification of fluorescence intensity at the injury site (n = 3/group). Mean values are shown and error bars represent ± s.d., as analyzed by one-way ANOVA with Tukey’s post hoc tests in (B). ns, not significant; ****P < 0.0001.


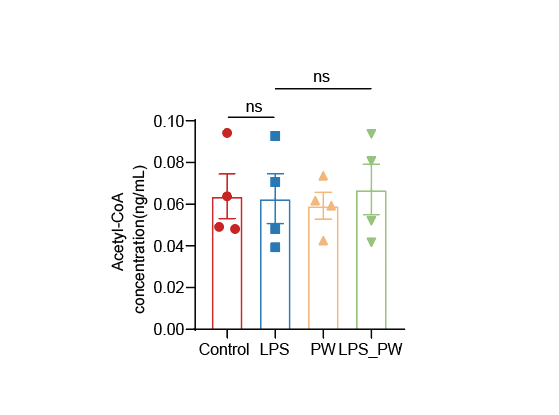


**Figure S18. Intracellular acetyl-CoA levels in BMDMs under different treatments.** Quantification of acetyl-CoA in BMDMs (n = 4/group). Mean values are shown and error bars represent ± s.d., as analyzed by one-way ANOVA with Tukey’s post hoc tests. ns, not significant.


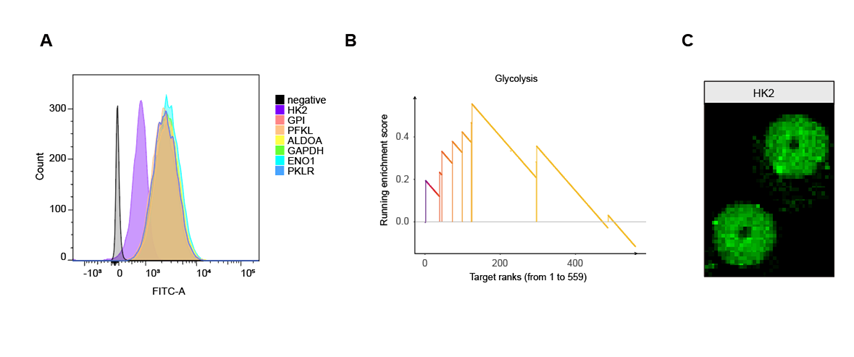


**Figure S19. PW targets glycolytic metabolism via HK2 binding. (A)** Flow cytometry analysis of glycolytic enzyme expression. **(B)** GSEA showing glycolysis pathway inhibition by PW. **(C)** Human Microarray Proteomic Analysis confirming PW binding to HK2.

**
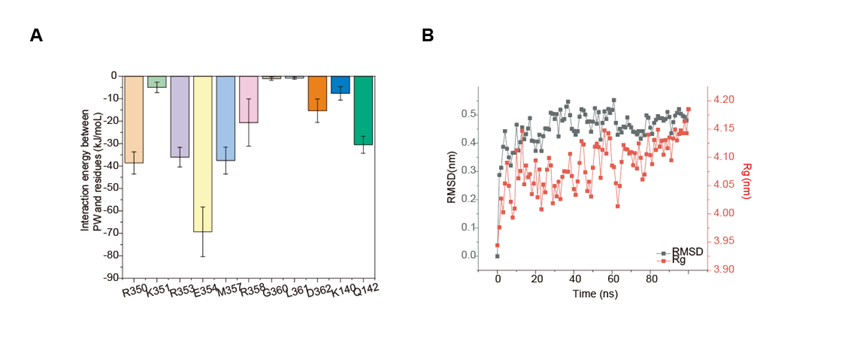
**

**Figure S20. Molecular dynamics simulation of PW–HK2 interaction. (A)** Interaction energy analysis of key binding residues. **(B)** Root mean square deviation (RMSD) and radius of gyration (Rg) over simulation time.


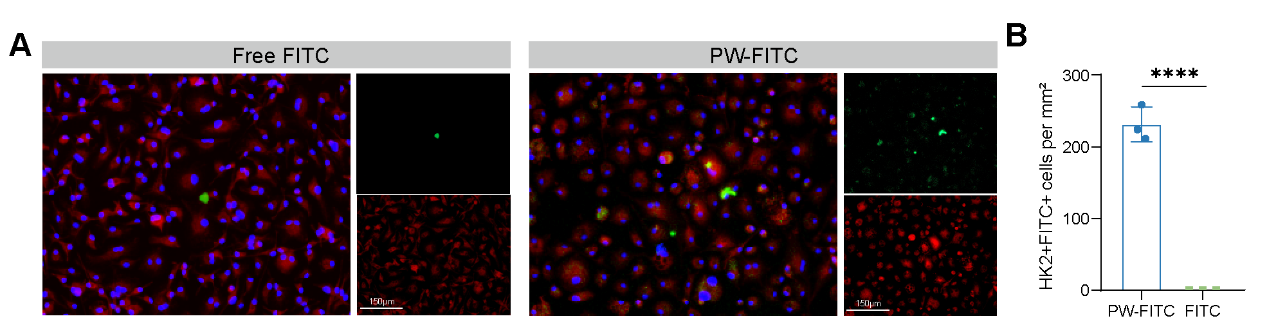


**Figure S21.** Intracellular co-localization of PW-FITC with HK2 in LPS-induced inflammatory macrophages. **(A)** Representative confocal images of LPS-induced inflammatory macrophages treated with free FITC or FITC-labeled PW (PW-FITC) for 12 h. HK2 was detected by immunofluorescence (red), PW-FITC or free FITC is shown in green, and nuclei were counterstained with DAPI (blue). Scale bars: 150 μm **(B)** Quantification of HK2^+^FITC^+^ cells per mm^2^, demonstrating significantly increased intracellular association of PW-FITC with HK2 compared to free FITC (n = 3/group). Mean values are shown and error bars represent ± s.d., as analyzed by unpaired Student’s t test in (**B**). **** P < 0.0001.


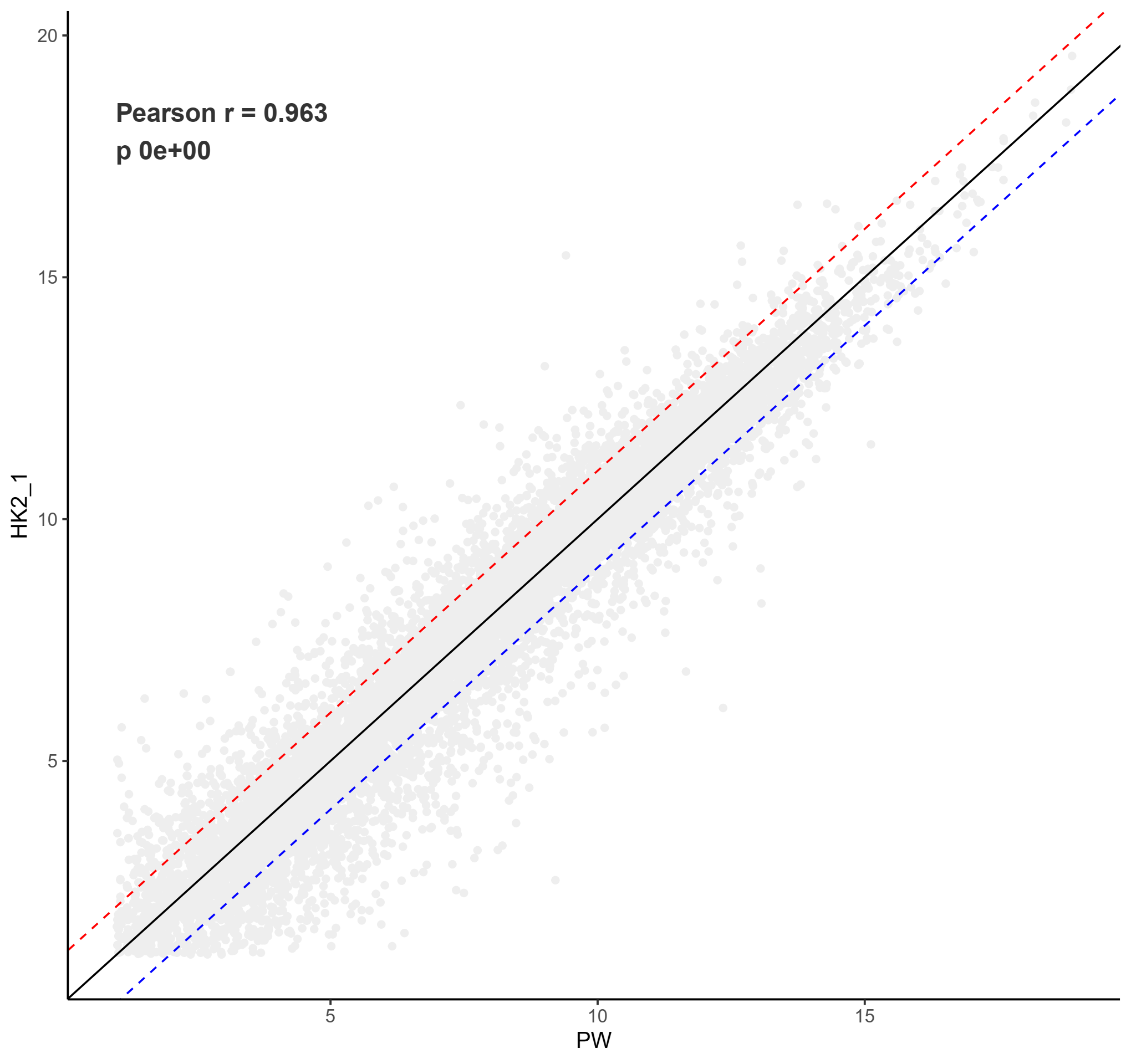


**Figure S22.** Global transcriptomic similarity analysis comparing PW versus PBS and HK2 inhibitor versus PBS.

**
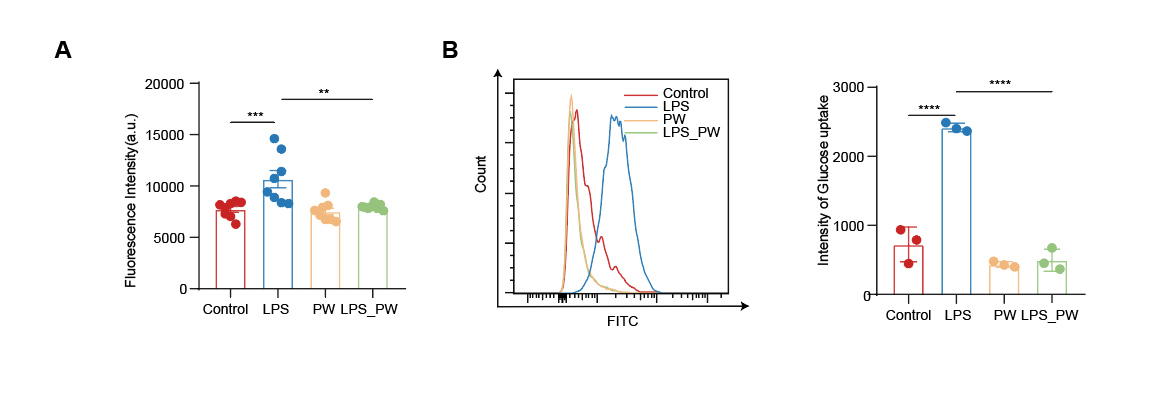
**

**Figure S23. PW inhibits glucose uptake in inflammatory BMDMs. (A)** Fluorescence intensity of glucose uptake (n = 8/group). **(B)** Flow cytometry analysis and quantification of glucose uptake (n = 3/group).


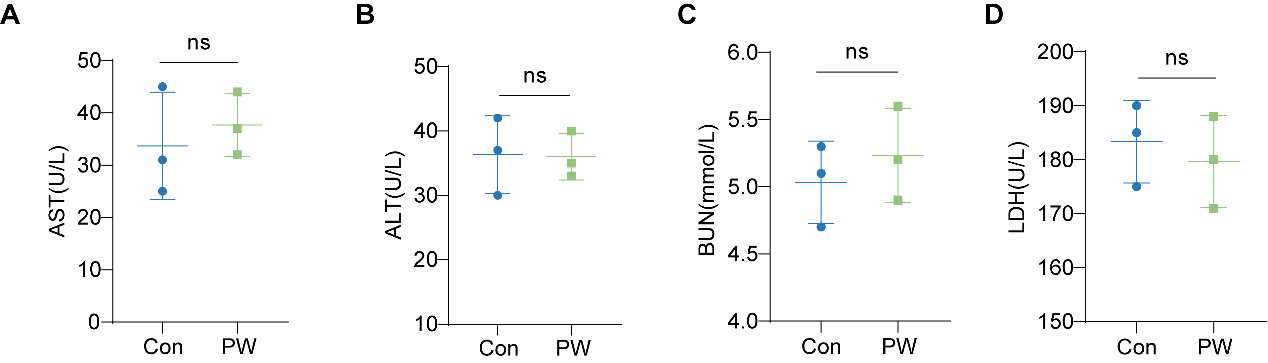


**Figure S24. Systemic safety assessment of PW in a canine model.** Serum levels of AST **(A)**, ALT **(B)**, LDH **(C)**, and BUN **(D)** in beagles 4 months post-treatment (n = 3/group). Mean values are shown and error bars represent ± s.d., as analyzed by t unpaired Student’s t test in (**A-D**). ns, not significant.
